# Commuting Quantum Operations Factorise

**DOI:** 10.1007/s00220-026-05655-9

**Published:** 2026-06-06

**Authors:** Renato Renner, Ramona Wolf

**Affiliations:** 1 Institute for Theoretical Physics, ETH Zurich, Zurich, Switzerland; 2 Quantum Center, ETH Zurich, Zurich, Switzerland; 3 Department of Theoretical Physics, University of Innsbruck, Innsbruck, Austria

## Abstract

Consider two agents, Alice and Bob, each of whom takes a quantum input, operates on a shared quantum system *K*, and produces a quantum output. Alice and Bob’s operations may commute, in the sense that the joint input-output behaviour is independent of the order in which they access *K*. Here we ask whether this commutation property implies that *K* can be split into two factors on which Alice and Bob act separately. The question can be regarded as a “fully quantum” generalisation of a problem posed by Tsirelson, who considered the case where Alice and Bob’s inputs and outputs are classical. In this case, the answer is negative in general, but it is known that a factorisation exists in finite dimensions. Here we show the same holds in the fully quantum case, i.e., commuting operations factorise, provided that all input systems are finite-dimensional.

## Introduction

Let *K* be a quantum system accessible to two agents, Alice and Bob, each operating on it once.^1^ Alice’s operation $\mathcal {X}_{I, A}$ depends on an input *I* and generates an output *A*. Similarly, Bob’s operation $\mathcal {Y}_{J, B}$ depends on *J* and generates *B*. We say that Alice and Bob’s operations *commute* if the order in which $\mathcal {X}_{I, A}$ and $\mathcal {Y}_{J, B}$ are applied has no influence on the outputs *A* and *B*, and on how they depend on the inputs *I* and *J*. Physical considerations often imply commutation. For example, it holds if Alice and Bob’s operations are executed at spacelike separation in a relativistic spacetime. Here we ask the following question:

*Does commutation between the maps* $\mathcal {X}_{I, A}$ *and* $\mathcal {Y}_{J, B}$ *imply the existence of a factorisation of* *K* *such that the maps act nontrivially solely on separate factors?*

**Fig. 1:**
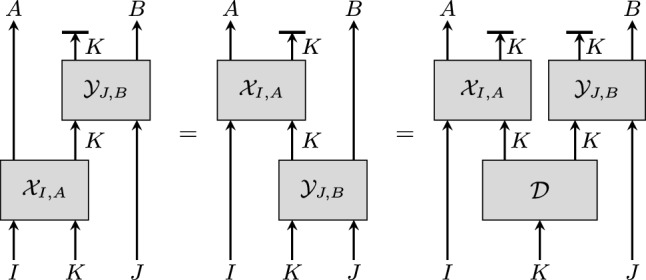
Illustration of the scenario described in the introduction. The circuit diagram corresponds to a special case of the diagram shown in Fig. 5 (see also Corollary 4.1). To see the correspondence, set $\mathcal {X}=\,\overline{\textrm{id}}_{I} \circ \mathcal {X}_{I, A} \otimes \mathcal {I}_J$ and $\mathcal {Y}=\mathcal {I}_I \otimes \mathcal {Y}_{J, B} \circ \,\overline{\textrm{id}}_{J}$, where $\,\overline{\textrm{id}}_{I}$ and $\,\overline{\textrm{id}}_{J}$ are completely positive maps that output a mixed state on *I* and *J*, respectively. The first two diagrams then match the first two diagrams of Fig. 5, where $H = I \otimes K \otimes J$. Similarly, the diagram to the right matches the diagram to the right of Fig. 5 for $\overline{\mathcal {X} }= {{\,\textrm{tr}\,}}_{K} \circ \mathcal {X}_{I, A}$, and $\overline{\mathcal {Y}} = {{\,\textrm{tr}\,}}_{K} \circ \mathcal {Y}_{J, B}$

This paper aims to provide a precise formulation of this question for the generic case where the inputs and outputs *I*, *J*, *A*, and *B* are arbitrary quantum systems (cf. Fig. 1). Our question can thus be understood as a “fully quantum” version of *Tsirelson’s problem*, which corresponds to the special case where these systems are all classical random variables (and these take values from finite sets). In the latter, $\mathcal {X}_{I, A}$ represents a measurement on *K* that depends on a classical choice, encoded in *I*, and outputs a classical result, encoded in *A*, and likewise for $\mathcal {Y}_{J, B}$. Tsirelson’s problem was initially posed in [14]. While it was prematurely claimed that it always has a positive answer, the proof, also by Tsirelson, assumes that *K* is finite-dimensional [15]. While this assumption can be relaxed [12], the answer to Tsirelson’s problem is negative if it is dropped completely [7] (see [2] for an overview).

The main technical contribution of this work is a proof that answers the general question above affirmatively under the assumptions that the map $\mathcal {X}_{I, A}$ or the map $\mathcal {Y}_{J, B}$ is unital on *K*, which is automatically satisfied in Tsirelson’s problem, and that the systems *I*, *J*, and *K* are finite-dimensional.

The paper is structured as follows: Section 2 outlines mathematical preliminaries. In Section 3, we present the main theorem of this work, Theorem 3.1, which provides sufficient conditions for two maps such that their action on a system *K* can be factorised. We then prove that these conditions are also necessary (Theorem 3.6). In Section 4, we show how our main theorem answers the question posed above (Corollary 4.1). In the same section, we demonstrate that Tsirelson’s answer to his question (phrased as Corollary 4.2) can be retrieved from Corollary 4.1. Finally, we present a generalisation of our main theorem to more than two parties operating on *K* (Corollary 4.5).

## Preliminaries

This section collects definitions, notation and theorems that are used in the proofs of the paper.

### Notation 2.1

We label Hilbert spaces with capital letters *H*, *K*, and so on. We also associate the same labels to the corresponding spaces of linear operators on these Hilbert spaces, and it should be clear from context which object is meant. We sometimes use the term *systems* to refer to these spaces. For example, we write $\rho _{H K}$ for a state (density operator) on the product of two systems *H* and *K*. Furthermore, we use the notation $\rho _H :={{\,\textrm{tr}\,}}_{K}(\rho _{H K})$, where ${{\,\textrm{tr}\,}}_K$ is the partial trace that acts on the space of linear operators on *K*.

### Notation 2.2

We denote by $\textrm{id}_H$ the identity operator on the Hilbert space *H*. For *H* finite-dimensional, $\,\overline{\textrm{id}}_{H}$ is the normalised maximally mixed state on *H*.

### Notation 2.3

We write $\mathcal {M}:H\rightarrow K$ or $\mathcal {M}_{H \rightarrow K}$ to indicate that a completely positive (CP) map $\mathcal {M}$ goes from a system *H* to a system *K*. That is, the map takes as input a trace-class operator on *H* and outputs a trace-class operator on *K*. For a CP map $\mathcal {M}_{H\rightarrow K R}$ we use the notation $\mathcal {M}_{H\rightarrow K} :=\textrm{tr}_R\circ \mathcal {M}_{H\rightarrow K R}$. We usually omit identity maps, i.e., $\mathcal {M}_{H\rightarrow K}(\rho _{HR}):=\left( \mathcal {M}_{H\rightarrow K}\otimes \mathcal {I}_{R}\right) (\rho _{H R})$.

### Remark 2.4

A CP map $\mathcal {M}: H \rightarrow K$ is trace-preserving (TP) if and only if ${{\,\textrm{tr}\,}}(\mathcal {M}(W_H)) = \textrm{tr}(W_H)$ holds for any trace-class operator $W_H$. This may also be written as

$$\begin{aligned} {{\,\textrm{tr}\,}}_K \circ \mathcal {M}_{H \rightarrow K} = {{\,\textrm{tr}\,}}_H. \end{aligned}$$

Note also that, if $\mathcal {M}$ has the Kraus representation $\mathcal {M}: \, W_H \mapsto \sum _z E_z W_H E_z^*$, then the TP property is equivalent to $\sum _z E_z^* E_z = \textrm{id}_H$.

Similarly, $\mathcal {M}$ is trace non-increasing if and only if ${{\,\textrm{tr}\,}}(\mathcal {M}(W_H)) \le \textrm{tr}(W_H)$ for any trace-class operator $W_H \ge 0$ or, equivalently, if the Kraus operators satisfy $\sum _z E_z^* E_z \le \textrm{id}_H$. Note that this also implies the operator inequality

$$\begin{aligned} {{\,\textrm{tr}\,}}_K \circ \mathcal {M}_{H \rightarrow K}(W_{H R})) \le {{\,\textrm{tr}\,}}_H(W_{H R}) \qquad \forall \, W_{H R} \ge 0. \end{aligned}$$

### Notation 2.5

For any state $\rho _H$, we can define a CPTP map from $\mathbb {C}$ to *H*, which takes a trivial (1-dimenstional) system as input and outputs $\rho _H$, i.e.,

$$\begin{aligned} W \mapsto W \rho _H. \end{aligned}$$

We denote this map also by $\rho _H$. Note that the concatenation ${{\,\textrm{tr}\,}}_H \circ \rho _H$ is equal to the identity map.

### Definition 2.6

A CP map $\mathcal {M}:H \rightarrow H$ is unital if $\mathcal {M}(\textrm{id}_H) = \textrm{id}_H$.

### Definition 2.7

A CP map $\mathcal {M}: H \otimes I \rightarrow K$ is independent of *I* if there exists a CP map $\overline{\mathcal {M}}: H\rightarrow K$ such that

1 $$\begin{aligned} \mathcal {M}_{HI\rightarrow K}=\overline{\mathcal {M}}_{H\rightarrow K}\circ \textrm{tr}_I. \end{aligned}$$

### Remark 2.8

If $\mathcal {M}: H \otimes I \rightarrow K$ is independent of *I* then the map $\overline{\mathcal {M}}_{H\rightarrow K}$ in (1) is unique and equal to the map $\mathcal {M}_{H I \rightarrow K} \circ \zeta _I$, i.e.,

$$\begin{aligned} \overline{\mathcal {M}}_{H\rightarrow K}:W_H \mapsto \mathcal {M}_{HI \rightarrow K}(W_H \otimes \zeta _I), \end{aligned}$$

where $\zeta _I$ is an arbitrary state on *I*.

### Lemma 2.9

For positive operators $\rho _{GH}$ and $\sigma _{KH}$ where $\rho _{GH}$ is pure and $\rho _H=\sigma _H$, there exists a CPTP map $\mathcal {R}: G \rightarrow K$ such that

$$\begin{aligned} \sigma _{KH}=\mathcal {R}_{G\rightarrow K}(\rho _{GH}). \end{aligned}$$

### Proof

Let $\tilde{\sigma }_{KEH}$ be a purification of $\sigma _{KH}$. The vector representations of the pure states $\rho _{GH}$ and $\tilde{\sigma }_{KEH}$ then have Schmidt decompositions $\sum _{i \in \mathfrak {I}} \lambda _i |g_i \rangle _{G} \otimes |h_i \rangle _H$ and $\sum _{i \in \mathfrak {I}} \lambda _i |e_i \rangle _{KE} \otimes |h_i \rangle _H$, where $\{|g_i \rangle _{G}\}_{i \in \mathfrak {I}}$, $\{|e_i \rangle _{KE}\}_{i \in \mathfrak {I}}$, and $\{|h_i \rangle _H\}_{i \in \mathfrak {I}}$ are orthonormal families of eigenvectors of $\rho _G$, $\tilde{\sigma }_{KE}$, and $\rho _H = \tilde{\sigma }_H$, respectively. By adding additional orthonormal vectors, we can extend $\{|g_i \rangle _{G}\}_{i \in \mathfrak {I}}$ to an orthonormal basis $\{|g_i \rangle _{G}\}_{i \in \mathfrak {I'}}$ of *G*.^2^ And because we can without loss of generality choose *K* such that the space *KE* is larger than *G*, we can also add orthonormal vectors to $\{|e_i \rangle _{KE}\}_{i \in \mathfrak {I}}$ to obtain a larger family $\{|e_i \rangle _{K E}\}_{i \in \mathfrak {I'}}$. We may now define an isometry *V* from *G* to *KE* by $|g_i \rangle _G \mapsto |e_i \rangle _{K E}$ for any $i \in \mathfrak {I'}$. Then, $\mathcal {R}_{G \rightarrow K E}: W_G \mapsto V W_G V^{*}$ is a CPTP map with the property $\tilde{\sigma }_{K E H}=\mathcal {R}_{G \rightarrow K E} (\rho _{G H})$. We thus have $\sigma _{KH}=\textrm{tr}_E \circ \mathcal {R}_{G\rightarrow K E}(\rho _{GH})$ as desired. $\square$

### Remark 2.10

We will make heavy use of the Choi-Jamiołkowski (C.-J.) isomorphism [4, 6], according to which a CP map $\mathcal {M}:H \rightarrow K$, where *H* has finite dimension, can be represented as a bipartite positive operator $\rho _{K \tilde{H}}:= \mathcal {M}(\Omega _{ H \tilde{H}})$, where $\Omega _{H\tilde{H}}:=|\Omega \rangle \!\langle \Omega |_{H\tilde{H}}$ is a maximally entangled state between *H* and an isomorphic system $\tilde{H}$. The C.-J. isomorphism depends on the choice of $\smash {\Omega _{H \tilde{H}}}$, which we will thus assume to be fixed. Note that, if *H* is composed of subsystems, then $\Omega _{H \tilde{H}}$ induces an analogous subsystem structure on $\tilde{H}$.

We will, in particular, consider spaces that decompose as $H = \bigoplus _z H_A^z \otimes H_B^z$. To reflect this decomposition on $\tilde{H}$, we equip *H* with an orthonormal basis of the form $\{ |a \rangle _{H^z_A} \otimes |b \rangle _{H^z_B}\}_{z, a, b}$, where, for any *z*, $\{|a \rangle _{H^z_A}\}_{a \in \mathfrak {A}^z}$ and $\{|b \rangle _{H^z_B}\}_{b \in \mathfrak {B}^z}$ are orthonormal bases of $H^z_A$ and $H^z_B$, respectively, and write the Schmidt decomposition of $|\Omega \rangle _{H \tilde{H}}$ as

$$\begin{aligned} |\Omega \rangle _{H \tilde{H}} = \sqrt{{\textstyle \frac{1}{\textrm{dim}(H)}}} \sum _{z} \sum _{\begin{array}{c} a \in \mathfrak {A}^z \\ b \in \mathfrak {B}^z \end{array}} \bigl ( |a \rangle _{H^z_A} \otimes |b \rangle _{H^z_B} \bigr ) \otimes |\varphi _{z, a, b} \rangle _{\tilde{H}}, \end{aligned}$$

where $|\varphi _{z, a, b} \rangle _{\tilde{H}}$ are appropriately chosen normalised vectors on $\tilde{H}$. We may now, for any fixed *z*, define the subspace $\tilde{H}^z :=\textrm{span} \{|\varphi _{z, a, b} \rangle \}_{a \in \mathfrak {A}^z, b \in \mathfrak {B}^z}$. Furthermore, we may introduce new spaces $\tilde{H}_A^z$ and $\tilde{H}_B^z$ with orthonormal bases $\{|a \rangle _{\tilde{H}^z_A}\}_{a \in \mathfrak {A}^z}$ and $\{|b \rangle _{\tilde{H}^z_B}\}_{b \in \mathfrak {B}^z}$, respectively, and define their tensor product by the bilinear map $\otimes : \, \tilde{H}_A^z \times \tilde{H}_B^z \rightarrow \tilde{H}^z$, which maps $(|a \rangle _{\tilde{H}^z_A}, |b \rangle _{\tilde{H}^z_B})$ to $|\varphi _{z, a, b} \rangle$, for any $a \in \mathfrak {A}^z, b \in \mathfrak {B}^z$. This definition ensures that $\smash {\tilde{H}^z = \tilde{H}_A^z \otimes \tilde{H}_B^z}$. The maximally entangled state $|\Omega \rangle _{H \tilde{H}}$ can then be expressed as

$$\begin{aligned} |\Omega \rangle _{H \tilde{H}} = \sqrt{{\textstyle \frac{1}{\textrm{dim}(H)}}} \sum _{z} \sum _{\begin{array}{c} a \in \mathfrak {A}^z \\ b \in \mathfrak {B}^z \end{array}} \bigl ( |a \rangle _{H^z_A} \otimes |b \rangle _{H^z_B} \bigr ) \otimes \bigl ( |a \rangle _{\tilde{H}^z_A} \otimes |b \rangle _{\tilde{H}^z_B} \bigr ) \end{aligned}$$

A special case of this is if *H* factorises into $H_A \otimes H_B$. Then there exists a factorisation of $\tilde{H}$ into $\tilde{H}_A \otimes \tilde{H}_B$ such that $|\Omega \rangle _{H \tilde{H}} = |\Omega \rangle _{H_A \tilde{H}_A} \otimes |\Omega \rangle _{H_B \tilde{H}_B}$, where $|\Omega \rangle _{H_A \tilde{H}_A}$ and $|\Omega \rangle _{H_B \tilde{H}_B}$ are maximally entangled states on $H_A \otimes \tilde{H}_A$ and $H_B \otimes \tilde{H}_B$, respectively.

We summarise some further basic properties of the C.-J. isomorphism (see Appendix A for proofs): The map $\mathcal {M}$ is TP if and only if $\rho _{\tilde{H}}=\,\overline{\textrm{id}}_{\tilde{H}}$. In this case $\rho _{K \tilde{H}}$ is normalised and thus a state. Furthermore, $\mathcal {M}$ is trace non-increasing if and only if $\rho _{\tilde{H}}\le \,\overline{\textrm{id}}_{\tilde{H}}$. $\mathcal {M}$ can be retrieved from $\rho _{K\tilde{H}}$ via

$$\begin{aligned} \mathcal {M}(W_H)=\dim (H)^2\,\textrm{tr}_{\tilde{H}}\Big (\textrm{tr}_H\big (W_H\Omega _{H\tilde{H}}\big )\rho _{K\tilde{H}}\Big ). \end{aligned}$$

### Lemma 2.11

Let $\mathcal {M}:H\rightarrow H$ be a unital, trace non-increasing CP map on a finite-dimensional space *H*. Then $\mathcal {M}$ is TP.

### Proof

According to Remark 2.10, a map $\mathcal {M}:H\rightarrow H$ is trace non-increasing if and only if its C.-J. operator $\rho _{H\tilde{H}}=\mathcal {M}(\Omega _{H\tilde{H}})$ fulfils

2 $$\begin{aligned} \rho _{\tilde{H}}=\textrm{tr}_H\rho _{H\tilde{H}}\le \,\overline{\textrm{id}}_{\tilde{H}}. \end{aligned}$$

Because $\mathcal {M}$ is unital, we also know that

$$\begin{aligned} \rho _H=\textrm{tr}_{\tilde{H}}\circ \mathcal {M}(\Omega _{H\tilde{H}})=\mathcal {M}\left( \,\overline{\textrm{id}}_{H}\right) =\,\overline{\textrm{id}}_{H}. \end{aligned}$$

The latter implies $\textrm{tr}(\rho _{\tilde{H}})=\textrm{tr}(\rho _{H\tilde{H}})=\textrm{tr}(\rho _H)=\textrm{tr}(\,\overline{\textrm{id}}_{H}) = \textrm{tr}(\,\overline{\textrm{id}}_{\tilde{H}})$. But this can only be true if the operator inequality (2) is an equality, i.e., $\rho _{\tilde{H}}=\,\overline{\textrm{id}}_{\tilde{H}}$. Hence, from the C.-J. isomorphism (see again Remark 2.10) it follows that $\mathcal {M}$ is TP. $\square$

### Definition 2.12

Let $\rho _{AB}$ be a density operator on $H_A\otimes H_B$. The conditional quantum entropy $H(A|B)_\rho$ is defined as

$$\begin{aligned} H(A|B)_\rho =H(AB)_\rho -H(B)_\rho \end{aligned}$$

with $$H(A)_\rho =-{{\,\textrm{tr}\,}}(\rho _A\log \rho _A)$$.

### Definition 2.13

Let $\rho _{ABC}$ be a density operator on $H_A\otimes H_B\otimes H_C$. The conditional mutual information $I(A:B|C)_\rho$ is defined as

$$\begin{aligned} I(A:B|C)_\rho =H(A|C)_\rho +H(B|C)_\rho -H(AB|C)_\rho . \end{aligned}$$

### Lemma 2.14

Let $\mathcal {M}:H \otimes I \rightarrow K$ be a CPTP map for finite-dimensional spaces *H*, *I*, *K* such that $\mathcal {M}$ is independent of *I*, and let $\rho _{K\tilde{H}\tilde{I}}$ be the C.-J. state of $\mathcal {M}$. Then

### Proof

Because $\mathcal {M}$ is independent of *I*, there exists a CPTP map $\overline{\mathcal {M}}:H\rightarrow K$ such that $\overline{\mathcal {M}}\circ \textrm{tr}_I=\mathcal {M}$. Then, for a maximally entangled state $\Omega _{HI\tilde{H}\tilde{I}}=\Omega _{H\tilde{H}} \otimes \Omega _{I\tilde{I}}$ (see Remark 2.10),

$$\begin{aligned} \rho _{K\tilde{H}\tilde{I}}&=\left( \mathcal {M}\otimes \mathcal {I}_{\tilde{H}\tilde{I}}\right) (\Omega _{HI\tilde{H}\tilde{I}})\\&=\left( \overline{\mathcal {M}}\circ \textrm{tr}_I\otimes \mathcal {I}_{\tilde{H}\tilde{I}}\right) (\Omega _{H\tilde{H}} \otimes \Omega _{I\tilde{I}})\\&=\left( \overline{\mathcal {M}}\otimes \mathcal {I}_{\tilde{H}} \otimes \mathcal {I}_{\tilde{I}}\right) \left( \Omega _{H\tilde{H}} \otimes \,\overline{\textrm{id}}_{\tilde{I}}\right) \\&=\left( \overline{\mathcal {M}}\otimes \mathcal {I}_{\tilde{H}}\right) (\Omega _{H\tilde{H}}) \otimes \,\overline{\textrm{id}}_{\tilde{I}}. \end{aligned}$$

From this tensor product structure of $\rho _{H\tilde{I}\tilde{R}}$, it follows that

$$\begin{aligned} H(K|\tilde{H}\tilde{I})_\rho&=H(K\tilde{H}\tilde{I})_\rho -H(\tilde{H}\tilde{I})_\rho \\&=H(K\tilde{H})_\rho +H(\tilde{I})_\rho -H(\tilde{H})_\rho -H(\tilde{I})_\rho \\&=H(K|\tilde{H})_\rho , \end{aligned}$$

hence . $$\square $$

### Lemma 2.15

For any CP map $\mathcal {M}:H\rightarrow \mathbb {C}$ there exists a Hermitian operator $M_H$ such that

$$\begin{aligned} \mathcal {M}(W_H)=\textrm{tr}(M_H W_H). \end{aligned}$$

### Proof

Let $\{E_z\}_z$ be the Kraus operators of $\mathcal {M}$, i.e., $\mathcal {M}: \, W_H \mapsto \sum _zE_z W_H E_z^*$. Because the image of $\mathcal {M}$ is one-dimensional, we have that $\mathcal {M}(W_H)=\textrm{tr}(\mathcal {M}(W_H))$. Hence, using cyclicity and linearity of the trace,

$$\begin{aligned} \mathcal {M}(W_H)&=\textrm{tr}\left( \sum _zE_z W_H E_z^*\right) =\textrm{tr}\left( \sum _zE_z^*E_z W_H\right) =\textrm{tr}(M W_H), \end{aligned}$$

where $$M=\sum _zE_z^*E_z$$. $$\square $$

### Remark 2.16

Let $\mathcal {M}:H\rightarrow K$ be a CP map. If *H* is finite-dimensional then

$$\begin{aligned} \lambda :=\sup _{\rho _H} {{\,\textrm{tr}\,}}(\mathcal {M}(\rho _H)), \end{aligned}$$

where the supremum ranges over all states on *H*, is finite. Hence, the rescaled map $\frac{1}{\lambda } \mathcal {M}$ is trace non-increasing.

### Lemma 2.17

Let $\mathcal {M}:H\rightarrow K$ be a trace non-increasing CP map and let $K' :=K\oplus \textrm{span}\{|\perp \rangle \}$, with $|\perp \rangle$ a unit vector. Then the map from *H* to $K'$ defined by^3^

3 $$\begin{aligned} \mathcal {M}' :=\mathcal {M} + \perp _{K'} \circ \bigl (\textrm{tr}_H - \textrm{tr}_{K} \circ \mathcal {M} \big ), \end{aligned}$$

is CP and TP. Furthermore, if $H= I \otimes J$ and $\mathcal {M}$ is independent of *I*, then $\mathcal {M}'$ is also independent of *I*.

### Proof

We start by showing the complete positivity of $\mathcal {M}'$. It suffices to verify that the map ${{\,\textrm{tr}\,}}_H - {{\,\textrm{tr}\,}}_K \circ \mathcal {M}$ is CP, which is equivalent to

$$\begin{aligned} {{\,\textrm{tr}\,}}_H(\rho _{H \tilde{H}}) \ge {{\,\textrm{tr}\,}}_K(\mathcal {M}(\rho _{H \tilde{H}})) \quad \forall \rho _{H \tilde{H}} \ge 0. \end{aligned}$$

This operator inequality follows from the assumption that $\mathcal {M}$ is trace non-increasing (see Remark 2.4). The map $\mathcal {M}'$ is also TP. This can be verified by taking the trace on the right-hand side of (3) and noting that ${{\,\textrm{tr}\,}}(\perp _{K'}) = 1$. Finally, the independence of $\mathcal {M}'$ from *I* can be verified by inspecting the right-hand side of (3), where the maps $\mathcal {M}$ and $\textrm{tr}_H$ are independent of *I*. $\square$

For the convenience of the reader we also state here Theorem 6 of [5], since this will be used in later proofs.

### Theorem 2.18

(Theorem 6 of [5]). Let *A*, *B*, *C* be finite-dimensional Hilbert spaces. A state $\rho _{ABC}$ on $A\otimes B\otimes C$ satisfies  if and only if there is a decomposition of *C* as

$$\begin{aligned} C=\bigoplus _z C_A^z\otimes C_B^z \end{aligned}$$

into a direct sum of tensor products, such that

$$\begin{aligned} \rho _{ABC}=\sum _z p_z\,\rho ^{z}_{AC_A^z}\otimes \rho ^{z}_{C_B^z B} \end{aligned}$$

with states $\rho ^{z}_{AC_A^z}$ on $A\otimes C_A^z$ and $\rho ^{z}_{C_B^z B}$ on $C_B^z\otimes B$, and a probability distribution $\{p_z\}$.

## Main Result

**Fig. 2:**
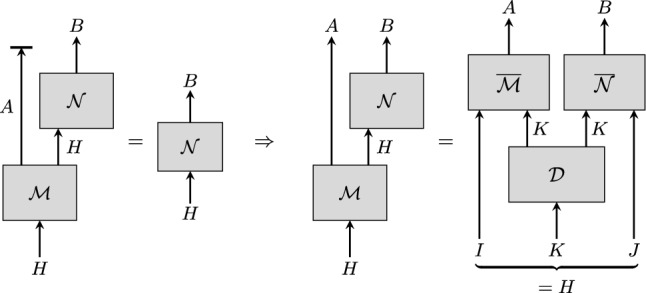
Visualisation of Theorem 3.1. The equality on the left-hand side illustrates Condition (i). Given that Conditions (ii) and (iii) are also satisfied, the theorem implies the equality between the circuit diagrams on the right-hand side

The claim that commuting operations factorise, as described informally in the introduction, is a corollary from a more general statement, Theorem 3.1, which we present and prove in the following. The setting considered by the theorem is illustrated by Fig. 2. At the end of the section we also give a converse statement, Theorem 3.6, which implies that the assumptions we make in Theorem 3.1 are necessary for factorisation.

### Theorem 3.1

Let $\mathcal {M}:H\rightarrow A\otimes H$ and $\mathcal {N}:H\rightarrow B$ be CP maps, where $H= I \otimes K\otimes J$ is finite-dimensional, such that

(i) $$\textrm{tr}_A\circ \mathcal {N}\circ \mathcal {M}=\mathcal {N}$$
(ii) $\textrm{tr}_A\circ \mathcal {M}$ is unital and trace non-increasing
(iii) $\textrm{tr}_H\circ \mathcal {M}$ is independent of *J* and $\mathcal {N}$ is independent of *I*.

Then there exists a completely positive, trace-preserving map $\mathcal {D}:K\rightarrow K\otimes K$ (“doubling map”) such that

4 $$\begin{aligned} \mathcal {N}\circ \mathcal {M}=\big (\overline{\mathcal {M}}\otimes \overline{\mathcal {N}}\big )\circ \mathcal {D}, \end{aligned}$$

where $$\overline{\mathcal {M}}\circ {{\,\textrm{tr}\,}}_J={{\,\textrm{tr}\,}}_H\circ \mathcal {M}$$, $$\overline{\mathcal {N}}\circ {{\,\textrm{tr}\,}}_I=\mathcal {N}$$.^4^

**Proof Outline.** The proof of Theorem 3.1 consists of the following steps:

(i) Let $\rho _{AB\tilde{H}}$ be the C.-J. operator of the map $\mathcal {N}\circ \mathcal {M}$, where $\tilde{H}$ is a Hilbert space isomorphic to *H*. Show that , i.e., $H(B\tilde{J}|\tilde{K})_{\rho }=H(B\tilde{J}|A\tilde{I}\tilde{K})_{\rho }$, via the data-processing inequality ($\rightarrow$ Claim 1).
(ii) Apply Theorem 2.18, which yields that $\rho _{AB\tilde{H}}$ is of the form
  $$\begin{aligned} \rho _{AB\tilde{H}}=\sum _z p_z\,\rho ^{z}_{A\tilde{I}\tilde{K}_A^z}\otimes \rho ^{z}_{\tilde{K}_B^z\tilde{J} B} \end{aligned}$$
  for a probability distribution $\{p_z\}$.
(iii) Show that $\rho _{AB\tilde{H}}$ above is equal to the C.-J. operator of the map $\big (\overline{\mathcal {M}}\otimes \overline{\mathcal {N}}\big )\circ \mathcal {D}$ $\rightarrow$ Claim 2).

### Proof of Theorem 3.1

We give the proof here under the assumption that the CP maps $\mathcal {M}$ and $\mathcal {N}$ are TP and that *A* and *B* are finite-dimensional. As we will explain in Remarks 3.2 and 3.4 below, these assumptions can be made without loss of generality.

First, we define a couple of quantum states that will be essential throughout the proof. Consider a Hilbert space $\tilde{H}$ that is isomorphic to *H*. The C.-J. operator of $\mathcal {N}\circ \mathcal {M}$ is given by

5 $$\begin{aligned} \rho _{AB\tilde{H}}=\mathcal {N}\circ \mathcal {M}(\Omega _{H \tilde{H}}), \end{aligned}$$

where $\Omega _{H\tilde{H}}:=|\Omega \rangle \!\langle \Omega |_{H\tilde{H}}$ is a maximally entangled state (see Remark 2.10). Thus, $\Omega _{H}=\,\overline{\textrm{id}}_{H}$ and $\Omega _{\tilde{H}}=\,\overline{\textrm{id}}_{\tilde{H}}$. Note that since we assume that $\mathcal {M}$ and $\mathcal {N}$ are TP, $\rho _{AB\tilde{H}}$ is normalised and hence a state.

**Fig. 3:**
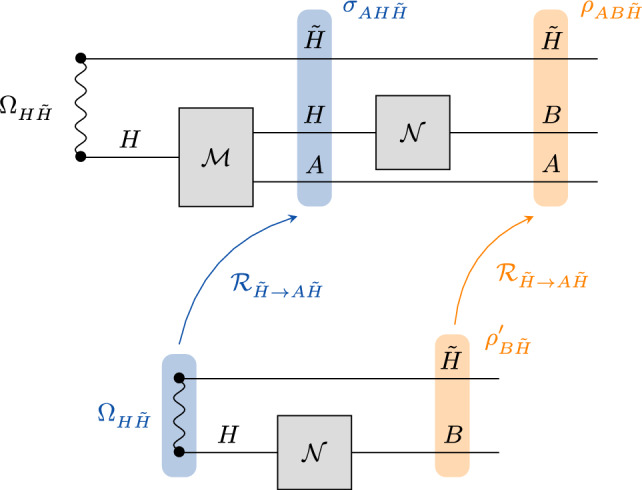
Relations between states used in the proof of Theorem 3.1. The diagram shows the states defined in (5) to (7) and the CP maps that connect them

We will furthermore need the following quantum states (see Fig. 3 for an illustration):

6 $$\begin{aligned} \sigma _{AH\tilde{H}}&=\mathcal {M}(\Omega _{H \tilde{H}})\end{aligned}$$

7 $$\begin{aligned} \rho '_{B\tilde{H}}&=\mathcal {N}(\Omega _{H\tilde{H}}). \end{aligned}$$

$$\square $$

### Claim 1

For the C.-J. operator $\rho _{AB\tilde{H}}$ of $\mathcal {N}\circ \mathcal {M}$ defined in (5), it holds that

### Proof of Claim 1

To prove the statement, we need to show that $H(B\tilde{J}|\tilde{K})_{\rho }=H(B\tilde{J}|A\tilde{I}\tilde{K})_{\rho }$. From strong subadditivity, it follows that

$$\begin{aligned} H(B\tilde{J}|A \tilde{I} \tilde{K})_{\rho }\le H(B\tilde{J}|\tilde{K})_{\rho }. \end{aligned}$$

To show the other direction, note that from the unitality of ${{\,\textrm{tr}\,}}_A\circ \mathcal {M}$ stated in Condition (ii), we have that

$$\begin{aligned} \begin{aligned} \sigma _H&=\textrm{tr}_{A\tilde{H}}\circ \mathcal {M}(\Omega _{H\tilde{H}})\\&=\textrm{tr}_{A}\circ \mathcal {M}\left( \,\overline{\textrm{id}}_{H}\right) \\&=\,\overline{\textrm{id}}_{H}\\&=\Omega _H, \end{aligned} \end{aligned}$$

i.e., $\Omega _{H\tilde{H}}$ is a purification of $\sigma _H$. From Lemma 2.9 with the assignment $G \rightarrow \tilde{H}$, $K \rightarrow A \otimes \tilde{H}$, $H \rightarrow H$, $\rho _{G H} \rightarrow \Omega _{\tilde{H} H}$, and $\sigma _{K H} \rightarrow \sigma _{A \tilde{H} H}$, we know there exists a CPTP map $\mathcal {R}_{\tilde{H}\rightarrow A\tilde{H}}$ such that the state in (6) can be written as

8 $$\begin{aligned} \sigma _{AH\tilde{H}}=\mathcal {R}_{\tilde{H}\rightarrow A\tilde{H}}(\Omega _{H\tilde{H}}). \end{aligned}$$

From (8), it follows that

9 $$\begin{aligned} \rho _{AB\tilde{H}}=\mathcal {N}(\sigma _{AH\tilde{H}})=\mathcal {N}\circ \mathcal {R}_{\tilde{H}\rightarrow A\tilde{H}}(\Omega _{H\tilde{H}})=\mathcal {R}_{\tilde{H}\rightarrow A\tilde{H}}\circ \mathcal {N}(\Omega _{H\tilde{H}})=\mathcal {R}_{\tilde{H}\rightarrow A\tilde{H}}(\rho '_{B\tilde{H}}), \end{aligned}$$

where we have used that $\mathcal {N}$ and $\mathcal {R}$ act on different systems and thus commute (see Fig. 3). With the chain rule for conditional entropy it follows thatIn the second line, because the map $\mathcal {N}$ is such that *B* is independent of *I*, we can apply Lemma 2.14, which yields $H(B|\tilde{K}\tilde{J})_{\rho '}=H(B|\tilde{K}\tilde{J}\tilde{I})_{\rho '}$. Also, we have used that $\rho '_{\tilde{H}}=\,\overline{\textrm{id}}_{\tilde{H}}$ implies $H(\tilde{J}|\tilde{K})_{\rho '}=H(\tilde{J}|\tilde{K}\tilde{I})_{\rho '}$. In the third line we have used (9) together with the data processing inequality, and in the fourth line we have used that $\rho _{\tilde{H}}=\,\overline{\textrm{id}}_{\tilde{H}}=\rho '_{\tilde{H}}$. The expression in the fifth line directly follows from the definition of the mutual information. The mutual information then vanishes because the map $\textrm{tr}_H \circ \mathcal {M}$ is such that *A* is independent of *J*, which allows us to apply Lemma 2.14. Finally, because of Condition (i), $\rho '_{B\tilde{H}}=\mathcal {N}(\Omega _{H\tilde{H}})=\textrm{tr}_A\circ \mathcal {N}\circ \mathcal {M}(\Omega _{H\tilde{H}})=\rho _{B\tilde{H}}$, hence it follows that

$$\begin{aligned} H(B\tilde{J}|\tilde{K})_{\rho '}=H(B\tilde{J}|\tilde{K})_{\rho }. \end{aligned}$$

Summarising, we have shown that

$$\begin{aligned} H(B\tilde{J}|\tilde{K}A \tilde{I})_{\rho }\le H(B\tilde{J}|\tilde{K})_{\rho }\le H(B\tilde{J}|\tilde{K}A \tilde{I} )_{\rho }, \end{aligned}$$

and therefore

$$\begin{aligned} H(B\tilde{J}|\tilde{K}A \tilde{I})_{\rho }= H(B\tilde{J}|\tilde{K})_{\rho }. \end{aligned}$$

Hence, , which is the statement of the claim.

$$\square $$

Using Theorem 2.18, Claim 1 implies that there exists a decomposition of $\tilde{K}$ of the form

$$\begin{aligned} \tilde{K}=\bigoplus _z \tilde{K}_A^z\otimes \tilde{K}_B^z \end{aligned}$$

such that

10 $$\begin{aligned} \rho _{AB\tilde{H}}=\sum _z p_z\,\rho _{A\tilde{I}\tilde{K}_A^z}^{z}\otimes \rho _{\tilde{K}_B^z\tilde{J} B}^{z}. \end{aligned}$$

Using the decomposition of $\tilde{K}$, we may decompose $\tilde{H}$ as

$$\begin{aligned} \tilde{H}&=\tilde{I}\otimes \tilde{K}\otimes \tilde{J} =\bigoplus _z\tilde{I}\otimes \tilde{K}_A^z\otimes \tilde{K}_B^z\otimes \tilde{J} =\bigoplus _z \tilde{A}^z\otimes \tilde{B}^z =\bigoplus _z \tilde{H}^z, \end{aligned}$$

where we have introduced the notation $\tilde{A}^z:=\tilde{I}\otimes \tilde{K}_A^z$, $\tilde{B}^z:=\tilde{K}_B^z\otimes \tilde{J}$, and $\tilde{H}^z:=\tilde{A}^z\otimes \tilde{B}^z$. We can thus rewrite (10) as

11 $$\begin{aligned} \rho _{AB\tilde{H}} = \sum _z p_z\,\rho _{A\tilde{A}^z}^{z}\otimes \rho _{\tilde{B}^z B}^{z} \ . \end{aligned}$$

Taking the trace over *A* and *B*, and applying a projection $\Pi _{\tilde{H}^z}$ onto $\tilde{H}^z$, for any *z*, we have

12 $$\begin{aligned} p_z\,\rho _{\tilde{A}^z}^{z}\otimes \rho _{\tilde{B}^z}^{z} = \Pi _{\tilde{H}^z}(\rho _{\tilde{H}}) = \Pi _{\tilde{H}^z}(\Omega _{\tilde{H}}) = \Pi _{\tilde{H}^z}(\,\overline{\textrm{id}}_{\tilde{H}}) \sim \textrm{id}_{\tilde{H}^z} = \textrm{id}_{\tilde{A}^z} \otimes \textrm{id}_{\tilde{B}^z}, \end{aligned}$$

where the second equality follows from (5) and the TP property of $\mathcal {M}$ and $\mathcal {N}$, and the third from the fact that $\Omega _{H \tilde{H}}$ is maximally entangled. To proceed, it will be convenient to introduce rescaled operators

$$\begin{aligned} \tau _{A\tilde{A}^z}^{z} \sim \rho _{A\tilde{A}^z}^{z} \qquad \text {and} \qquad \tau _{\tilde{B}^z B}^{z} \sim \rho _{\tilde{B}^z B}^{z}, \end{aligned}$$

which are normalised such that

13 $$\begin{aligned} \textrm{tr}\bigl ( \tau _{A\tilde{A}^z}^{z} \bigr ) = \dim (\tilde{A}^z) \qquad \text {and} \qquad \textrm{tr} \bigl ( \tau _{\tilde{B}^z B}^{z} \bigr ) = \dim (\tilde{B}^z). \end{aligned}$$

It then follows from (12) that

$$\begin{aligned} \tau _{\tilde{A}^z}^{z} = \textrm{id}_{\tilde{A}^z} \qquad \text {and} \qquad \tau _{\tilde{B}^z}^{z} = \textrm{id}_{\tilde{B}^z}. \end{aligned}$$

With these operators, we may rewrite (11) as

14 $$\begin{aligned} \rho _{AB\tilde{H}}=\sum _z q_z \tau _{A\tilde{A}^z}^{z}\otimes \tau _{\tilde{B}^z B}^{z} \end{aligned}$$

for some appropriately chosen weights $q_z$, which we will now determine. For this we again take the trace over *A* and *B* on both sides and apply the projection $\Pi _{\tilde{H}^z}$, which yields

$$\begin{aligned} \Pi _{\tilde{H}^z}(\rho _{\tilde{H}}) = q_z\,\tau ^{z}_{\tilde{A}^z}\otimes \tau ^{z}_{\tilde{B}^z} = q_z\,\textrm{id}_{\tilde{A}^z}\otimes \textrm{id}_{\tilde{B}^z} = q_z\,\textrm{id}_{\tilde{H}^z}. \end{aligned}$$

Since, according to (12), this must also equal $\Pi _{\tilde{H}^z}(\,\overline{\textrm{id}}_{\tilde{H}})$, we find

$$\begin{aligned} q_z\,\textrm{id}_{\tilde{H}^z} = \Pi _{\tilde{H}^z}(\,\overline{\textrm{id}}_{\tilde{H}}) = {\textstyle \frac{1}{\dim (H)}} \Pi _{\tilde{H}^z}(\textrm{id}_{\tilde{H}}) = {\textstyle \frac{1}{\dim (H)}} \textrm{id}_{\tilde{H}^z}, \end{aligned}$$

which implies $q_z=\frac{1}{\dim (H)}$. Inserting this into (14), we conclude that

15 $$\begin{aligned} \rho _{AB\tilde{H}} = {\textstyle \frac{1}{\dim (H)}} \sum _z \tau _{A\tilde{A}^z}^{z}\otimes \tau _{\tilde{B}^z B}^{z}. \end{aligned}$$

### Claim 2

There exists a CPTP map $\mathcal {D}:K\rightarrow K\otimes K$ such that

$$\begin{aligned} \mathcal {N}\circ \mathcal {M}=\big (\overline{\mathcal {M}}\otimes \overline{\mathcal {N}}\big )\circ \mathcal {D}, \end{aligned}$$

where $$\overline{\mathcal {M}}\circ {{\,\textrm{tr}\,}}_J={{\,\textrm{tr}\,}}_H\circ \mathcal {M}$$, $$\overline{\mathcal {N}}\circ {{\,\textrm{tr}\,}}_I=\mathcal {N}$$.

### Proof of Claim 2

In the following, we use the notation $\mathcal {D}:K\rightarrow K'\otimes K''$, where $K'=K=K''$, to make clear how the involved maps are acting on the different Hilbert spaces. Let

16 $$\begin{aligned} \mathcal {D}(W_K):=\sum _z V^{(z)}W_K {V^{(z)}}^*\otimes \,\overline{\textrm{id}}_{K_B'^z}\otimes \,\overline{\textrm{id}}_{K_A''^z}, \end{aligned}$$

where

17 $$\begin{aligned} V^{(z)}:=\sum _{a,b}\left( |a \rangle _{K_A'^z}\otimes |b \rangle _{K_B''^z}\right) \left( \langle a |_{K_A^z}\otimes \langle b |_{K_B^z}\right) . \end{aligned}$$

The map $\mathcal {D}$ is CP because each term in its definition is CP, and we will verify at the end of the proof that it is also TP.

Next, we calculate the C.-J. operator $\xi _{AB\tilde{H}}$ of the map $\big (\overline{\mathcal {M}}\otimes \overline{\mathcal {N}}\big )\circ \mathcal {D}$ with respect to the same state $\Omega _{H \tilde{H}} = |\Omega \rangle \!\langle \Omega |_{H \tilde{H}}$ as in (5). Because, according to Remark 2.10, this state can be written as $|\Omega \rangle _{H \tilde{H}} = |\Omega \rangle _{I \tilde{I}} \otimes |\Omega \rangle _{K \tilde{K}} \otimes |\Omega \rangle _{J \tilde{J}}$, where

18 $$\begin{aligned} |\Omega \rangle _{K\tilde{K}}={\textstyle \sqrt{\frac{1}{\dim (K)}}} \sum _{z,a,b} |a \rangle _{K_A^z}\otimes |b \rangle _{K_B^z} \otimes |a \rangle _{\tilde{K}_A^z} \otimes |b \rangle _{\tilde{K}_B^z}, \end{aligned}$$

we find

19 $$\begin{aligned} \xi _{AB\tilde{H}}&:=\big (\overline{\mathcal {M}}\otimes \overline{\mathcal {N}}\big )\circ \mathcal {D}\left( \Omega _{H\tilde{H}}\right) \nonumber \\&=\big (\overline{\mathcal {M}}\otimes \overline{\mathcal {N}}\big )\circ \mathcal {D}\left( |\Omega \rangle \!\langle \Omega |_{I\tilde{I}}\otimes |\Omega \rangle \!\langle \Omega |_{K\tilde{K}}\otimes |\Omega \rangle \!\langle \Omega |_{J\tilde{J}}\right) \nonumber \\&=\big (\overline{\mathcal {M}}\otimes \overline{\mathcal {N}}\big )\Bigg ({\textstyle \frac{1}{\dim (K)}} \sum _z\sum _{\begin{array}{c} a,b,\nonumber \\ \bar{a},\bar{b} \end{array}}\Bigl (|a \rangle \!\langle \bar{a} |_{K_A'^z} \otimes |b \rangle \!\langle \bar{b} |_{K_B''^z}\Bigr )\otimes \Bigl (|a \rangle \!\langle \bar{a} |_{\tilde{K}_A^z} \otimes |b \rangle \!\langle \bar{b} |_{\tilde{K}_B^z}\Bigr )\nonumber \\&\hspace{20pt}\otimes \,\overline{\textrm{id}}_{K_B'^z}\otimes \,\overline{\textrm{id}}_{K_A''^z}\otimes |\Omega \rangle \!\langle \Omega |_{I\tilde{I}}\otimes |\Omega \rangle \!\langle \Omega |_{J\tilde{J}}\Bigg )\nonumber \\&= {\textstyle \frac{1}{\dim (K)}} \sum _z\xi _{A\tilde{A}^z }^{z}\otimes \xi _{B\tilde{B}^z}^{z}, \end{aligned}$$

where

$$\begin{aligned} \xi _{A\tilde{A}^z}^{z} = \xi _{A\tilde{K}_A^z\tilde{I}}^{z}&:=\overline{\mathcal {M}}\left( \sum _{a,\bar{a}}|a \rangle \!\langle \bar{a} |_{K_A'^z}\otimes \,\overline{\textrm{id}}_{K_B'^z}\otimes |a \rangle \!\langle \bar{a} |_{\tilde{K}_A^z} \otimes |\Omega \rangle \!\langle \Omega |_{I\tilde{I}} \right) \\ \xi _{B\tilde{B}^z}^{z} = \xi _{B\tilde{K}_B^z\tilde{J}}^{z}&:=\overline{\mathcal {N}}\left( \sum _{b,\bar{b}}\,\overline{\textrm{id}}_{K_A''^z}\otimes |b \rangle \!\langle \bar{b} |_{K_B''^z}\otimes |b \rangle \!\langle \bar{b} |_{\tilde{K}_B^z} \otimes |\Omega \rangle \!\langle \Omega |_{J\tilde{J}} \right) . \end{aligned}$$

It remains to show that $\xi _{AB\tilde{H}}$ equals the C.-J. state $\rho _{AB\tilde{H}}$ of the map $\mathcal {N} \circ \mathcal {M}$. To this aim, we note that (13) implies

20 $$\begin{aligned} {{\,\textrm{tr}\,}}_{B\tilde{B}^z} \circ \Pi _{\tilde{H}^z} (\rho _{AB\tilde{H}}) = {\textstyle \frac{\dim (\tilde{B}^z)}{\dim (H)}} \, \tau _{A\tilde{A}^z}={\textstyle \frac{\dim (K_B^z)}{\dim (K)\dim (I)}} \, \tau _{A\tilde{A}^z}. \end{aligned}$$

Furthermore, because $\mathcal {N}$ is TP, we have ${{\,\textrm{tr}\,}}_B\circ \mathcal {N}\circ \mathcal {M}={{\,\textrm{tr}\,}}_H\circ \mathcal {M}$ (see Remark 2.4). It thus follows that the partial trace $\rho _{A\tilde{H}} = \textrm{tr}_B(\rho _{A B \tilde{H}})$ is the C.-J. state of ${{\,\textrm{tr}\,}}_H\circ \mathcal {M}$, and thus

$$\begin{aligned} \rho _{A\tilde{H}}&=\left( {{\,\textrm{tr}\,}}_H\circ \mathcal {M}\right) \left( |\Omega \rangle \!\langle \Omega |_{I\tilde{I}}\otimes |\Omega \rangle \!\langle \Omega |_{K\tilde{K}}\otimes |\Omega \rangle \!\langle \Omega |_{J\tilde{J}}\right) \\&=\left( \overline{\mathcal {M}}\circ {{\,\textrm{tr}\,}}_J\right) \left( |\Omega \rangle \!\langle \Omega |_{I\tilde{I}}\otimes |\Omega \rangle \!\langle \Omega |_{K\tilde{K}}\otimes |\Omega \rangle \!\langle \Omega |_{J\tilde{J}}\right) \\&=\overline{\mathcal {M}}\left( |\Omega \rangle \!\langle \Omega |_{I\tilde{I}}\otimes |\Omega \rangle \!\langle \Omega |_{K\tilde{K}}\right) \otimes \,\overline{\textrm{id}}_{\tilde{J}}. \end{aligned}$$

Using again (18), we can thus write

$$\begin{aligned}&{{\,\textrm{tr}\,}}_{B\tilde{B}^z} \circ \Pi _{\tilde{H}^z}(\rho _{AB\tilde{H}})\\&\quad ={{\,\textrm{tr}\,}}_{\tilde{B}^z} \circ \Pi _{\tilde{H}^z}(\rho _{A\tilde{H}})\\&\quad = {{\,\textrm{tr}\,}}_{\tilde{K}_B^z} \circ \Pi _{\tilde{H}^z} \circ \overline{\mathcal {M}}\left( |\Omega \rangle \!\langle \Omega |_{K\tilde{K}} \otimes |\Omega \rangle \!\langle \Omega |_{I\tilde{I}} \right) \\&\quad ={{\,\textrm{tr}\,}}_{\tilde{K}_B^z} \circ \overline{\mathcal {M}}\Bigg ({\textstyle \frac{1}{\dim (K)}} \sum _{\begin{array}{c} a,b,\\ \bar{a},\bar{b} \end{array}} |a \rangle \!\langle \bar{a} |_{K_A^{z}} \otimes |b \rangle \!\langle \bar{b} |_{K_B^{z}} \otimes |a \rangle \!\langle \bar{a} |_{\tilde{K}_A^{z}} \otimes |b \rangle \!\langle \bar{b} |_{\tilde{K}_B^{z}}\otimes |\Omega \rangle \!\langle \Omega |_{I\tilde{I}}\Bigg )\\&\quad ={\textstyle \frac{1}{\dim (K)}}\,\overline{\mathcal {M}}\Bigg (\sum _{a,\bar{a}}|a \rangle \langle \bar{a} |_{K_A^{z}}\otimes \underbrace{\textrm{id}_{K_B^z}}_{=\dim (K_B^z)\,\overline{\textrm{id}}_{K_B^z}}\otimes |a \rangle \!\langle \bar{a} |_{\tilde{K}_A^z}\otimes |\Omega \rangle \!\langle \Omega |_{I\tilde{I}}\Bigg )\\&\quad ={\textstyle \frac{\dim (K_B^z)}{\dim (K)}}\,\xi _{A\tilde{A}^z}^{z}. \end{aligned}$$

Comparing this to (20) yields

$$\begin{aligned} \xi _{A\tilde{A}^z}^{z}&={\textstyle \frac{1}{\dim (I)}} \, \tau _{A\tilde{A}^z}^{z}. \end{aligned}$$

By an analogous argument we obtain

$$\begin{aligned} \xi _{B\tilde{B}^z}^{z}&={\textstyle \frac{1}{\dim (J)}} \, \tau _{B\tilde{B}^z}^{z}. \end{aligned}$$

Inserting this into (19) and comparing to (15) yields $\xi _{AB\tilde{H}}=\rho _{AB\tilde{H}}$. Thus, the respective C.-J. states of $\mathcal {N}\circ \mathcal {M}$ and $(\overline{\mathcal {M}}\otimes \overline{\mathcal {N}})\circ \mathcal {D}$ are equal, hence the two maps are equal.

To conclude the proof of Claim 2, we need to verify that the map $\mathcal {D}$ is TP as claimed. Note first that, by the definition of $\overline{\mathcal {M}}$ and the TP property of $\mathcal {M}$ (see Remark 2.4),

$$\begin{aligned} \textrm{tr}_A \circ \overline{\mathcal {M}} \circ \textrm{tr}_J = \textrm{tr}_{A H} \circ \mathcal {M} = \textrm{tr}_H = \textrm{tr}_{K I J}. \end{aligned}$$

This implies that $\textrm{tr}_A \circ \overline{\mathcal {M}} = \textrm{tr}_{K I}$, which means that $\overline{\mathcal {M}}$ is TP. Similarly, one can see that $\overline{\mathcal {N}}$ is TP. Hence, $\overline{\mathcal {M}} \otimes \overline{\mathcal {N}}$, which goes from $K' \otimes K'' \otimes I \otimes J$ to $A \otimes B$, is TP, too. Using this, then (4), and finally that $\mathcal {N} \circ \mathcal {M}$ is TP, we find

$$\begin{aligned} \textrm{tr}_{K' K'' I J} \circ \mathcal {D} = \textrm{tr}_{A B} \circ (\overline{\mathcal {M}} \otimes \overline{\mathcal {N}}) \circ \mathcal {D} = \textrm{tr}_{A B} \circ \mathcal {N} \circ \mathcal {M} = \textrm{tr}_{K I J}. \end{aligned}$$

This implies that $\textrm{tr}_{K' K''} \circ \mathcal {D} = \textrm{tr}_{K}$, i.e., $\mathcal {D}$ is TP. $\square$

With the proof of Claim 2, we have established (4). $\square$

### Remark 3.2

Theorem 3.1 follows from a more specialised version of the same where the maps $\mathcal {M}$ and $\mathcal {N}$ are assumed to be TP. To see this, note first that Lemma 2.11 immediately implies that ${{\,\textrm{tr}\,}}_A \circ \mathcal {M}$ is TP. Hence, $\mathcal {M}$ must be TP anyway. It thus remains to show the following: For any map $\mathcal {N}$ from *H* to *B*, there exists a TP map $\mathcal {N}'$ such that the correctness of Theorem 3.1 for $\mathcal {N}'$ implies the correctness of the theorem for $\mathcal {N}$.

Let thus $\mathcal {N}$ be a CP map that satisfies the assumptions of Theorem 3.1. From Remark 2.16 and the fact that *H* is finite-dimensional, we know that $\mathcal {N}$ can always be rescaled such that it is trace non-increasing. The rescaling does not alter Condition (i), Condition (iii), and (4). We can thus assume without loss of generality that $\mathcal {N}$ is trace non-increasing. Let now $\mathcal {N}'$ be the TP extension of $\mathcal {N}$ defined by Lemma 2.17, which maps from *H* to $B'$, where $B' = B \oplus \textrm{span} \{|\perp \rangle \}$. We have

$$\begin{aligned} \textrm{tr}_{A}\circ \mathcal {N}' \circ \mathcal {M}&=\textrm{tr}_{A}\circ \,\mathcal {N}\circ \mathcal {M}+ \perp _{B'} \circ \big ( \textrm{tr}_A \circ \textrm{tr}_H \circ \mathcal {M}-\, \textrm{tr}_A \circ \textrm{tr}_B \circ \mathcal {N} \circ \mathcal {M} \big ) \\&=\mathcal {N}+ \perp _{B'} \circ \big (\textrm{tr}_H -\textrm{tr}_B \circ \mathcal {N} \big ) =\mathcal {N}'. \end{aligned}$$

where the second equality holds because $\mathcal {N}$ satisfies Condition (i), and because $\mathcal {M}$ is TP. This shows that $\mathcal {N}'$ also satisfies Condition (i). Furthermore, because $\mathcal {N}$ satisfies Condition (iii) by assumption, it is independent of *I*, and hence Lemma 2.17 implies the same is true for $\mathcal {N}'$. We have thus established that $\mathcal {N}'$ is a CPTP map that meets all conditions of Theorem 3.1. The specialised version of Theorem 3.1 for TP maps now implies that there exists a CPTP map $\mathcal {D}$ such that

$$\begin{aligned} \mathcal {N}' \circ \mathcal {M} = \left( \overline{\mathcal {M}} \otimes \overline{\mathcal {N}'}\right) \circ \mathcal {D}. \end{aligned}$$

We can concatenate both sides with a projection map $\Pi _B$ onto the subspace *B* of $B'$. Since $\mathcal {N} = \Pi _B\circ \mathcal {N}'$ and $\overline{\mathcal {N}} = \Pi _B\circ \overline{\mathcal {N}'}$ we find that (4) and, hence, Theorem 3.1, holds for the general map $\mathcal {N}$.

### Remark 3.3

The unitality requirement in Condition (ii) can be replaced by the weaker condition that $\textrm{tr}_A \circ \mathcal {M}$ is unital when restricted to the subsystem $K \otimes J$ of *H*, i.e.,

21 $$\begin{aligned} \textrm{tr}_{A I} \circ \mathcal {M}(\rho _{I} \otimes {{\,\textrm{id}\,}}_{K J}) = {{\,\textrm{id}\,}}_{K J}. \end{aligned}$$

To see this, let $\mathcal {M}$ be such that it satisfies Conditions (i) and (iii), as well as (21). Furthermore, define a modified map $\mathcal {M'} :=\,\overline{\textrm{id}}_{I} \circ {{\,\textrm{tr}\,}}_I \circ \mathcal {M}$. Because $\mathcal {N}$ is independent of *I*, $\mathcal {M'}$ also satisfies Condition (i). Clearly, it also satisfies Condition (iii). And because of (21), $\mathcal {M'}$ also fulfils Condition (ii). We can thus apply the theorem to the modified map $\mathcal {M'}$, which implies that (4) holds for $\mathcal {M}'$ and $\overline{\mathcal {M'}}$. But $\overline{\mathcal {M'}} = \overline{\mathcal {M}}$, which can be verified by using Remark 2.8:

$$\begin{aligned} \overline{\mathcal {M}'}&={{\,\textrm{tr}\,}}_H\circ \mathcal {M}'\circ \zeta _J\\&={{\,\textrm{tr}\,}}_H\circ \,\overline{\textrm{id}}_{I}\circ {{\,\textrm{tr}\,}}_I\circ \mathcal {M}\circ \zeta _J\\&={{\,\textrm{tr}\,}}_H\circ \mathcal {M}\circ \zeta _J\\&=\overline{\mathcal {M}}, \end{aligned}$$

where $\zeta _J$ is the map that creates a state $\zeta _J$ on *J*. Furthermore, again because $\mathcal {N}$ is independent of *I* and can thus be written as $\overline{\mathcal {N}}\circ {{\,\textrm{tr}\,}}_I$,

$$\begin{aligned} \mathcal {N}\circ \mathcal {M}'&=\overline{\mathcal {N}}\circ {{\,\textrm{tr}\,}}_I\circ \,\overline{\textrm{id}}_{I}\circ {{\,\textrm{tr}\,}}_I\circ \mathcal {M}\\&=\overline{\mathcal {N}}\circ {{\,\textrm{tr}\,}}_I\circ \mathcal {M}\\&=\mathcal {N}\circ \mathcal {M}. \end{aligned}$$

Hence, (4) also holds for $\mathcal {M}$.

### Remark 3.4

It is sufficient to prove Theorem 3.1 for finite-dimensional Hilbert spaces *A* and *B*, as this implies the general case where these systems have unbounded dimensions.

To see this, let $\mathcal {M}$ and $\mathcal {N}$ be CP maps for infinite-dimensional *A* and *B* that satisfy Conditions (i), (ii), and (iii) of Theorem 3.1. Furthermore, let $(\Pi _A^d)_{d \in \mathbb {N}}$ be a sequence of CP maps that project on *d*-dimensional nested subspaces of system *A*, i.e., $\Pi _A^d \circ \Pi _A^{d'} = \Pi _A^{d}$ for any $d \le d'$ such that, for all states $\rho _A$,

22 $$\begin{aligned} \lim _{d \rightarrow \infty } \Pi ^d_A(\rho _A) = \rho _A \end{aligned}$$

Similarly, we denote by $(\Pi _B^d)_{d \in \mathbb {N}}$ a sequence of projection maps for the system *B*. We can then define sequences of CP maps $(\mathcal {M}^d)_{d \in \mathbb {N}}$ and $(\mathcal {N}^d)_{d \in \mathbb {N}}$ by

$$\begin{aligned} \mathcal {M}^d&:=\bigl ( \Pi _A^d + \zeta _A \circ \textrm{tr}_A \circ (\mathcal {I}_A - \Pi _A^d)\bigr ) \circ \mathcal {M} \\ \mathcal {N}^d&:=\Pi _B^d \circ \mathcal {N}, \end{aligned}$$

where $\zeta _A$ is an arbitrary state on *A*.

When considering the convergence of sequences of CP maps, we use the topology of their C.-J. representation as states, which in turn is induced by the trace distance.^5^ Thus, using (22),

23 $$\begin{aligned} \lim _{d \rightarrow \infty } \mathcal {M}^d&= \mathcal {M} \end{aligned}$$

24 $$\begin{aligned} \lim _{d \rightarrow \infty } \mathcal {N}^d&= \mathcal {N} . \end{aligned}$$

Furthermore, for any $d \in \mathbb {N}$ we have

$$\begin{aligned} \textrm{tr}_A \circ \mathcal {M}^d = \textrm{tr}_A \circ \Pi _A^d \circ \mathcal {M} + \underbrace{\textrm{tr}_A(\zeta _A)}_{=1} (\textrm{tr}_A \circ \mathcal {M} - \textrm{tr}_A \circ \Pi _A^d \circ \mathcal {M}) = \textrm{tr}_A \circ \mathcal {M}. \end{aligned}$$

Using this one can readily verify that each of the pairs of maps $\mathcal {M}^d$ and $\mathcal {N}^d$ satisfies Conditions (i), (ii), and (iii). Theorem 3.1 thus implies that there exists a CPTP map $\mathcal {D}^d$ such that

25 $$\begin{aligned} \mathcal {N}^d \circ \mathcal {M}^d = \bigl ( \overline{\mathcal {M}^d} \otimes \overline{\mathcal {N}^d} \bigr ) \circ \mathcal {D}^d. \end{aligned}$$

Note that $\mathcal {D}^d$ is a CPTP map from *K* to $K \otimes K$, which is finite-dimensional. By the C.-J. isomorphism, the set of such maps is isomorphic to a closed subset of (normalised) states on a finite-dimensional space, which one may also purify. Furthermore, the set of pure states can be continuously embedded into a (real) Euclidean space, where it corresponds to a sphere of radius 1. Hence, the set of possible maps $\mathcal {D}^d$ is bounded and closed. We can thus employ the Bolzano-Weierstrass theorem, which tells us that there exists a subsequence of $(\mathcal {D}^d)_{d \in \mathbb {N}}$ that converges to a CPTP map $\mathcal {D}$, i.e., there exists $(d_i)_{i \in \mathbb {N}}$ such that

$$\begin{aligned} \mathcal {D} = \lim _{i \rightarrow \infty } \mathcal {D}^{d_i} \ . \end{aligned}$$

Note that the convergence of the sequences (23) and (24) also implies the convergence of the subsequences, i.e., $\lim _{i \rightarrow \infty } \mathcal {M}^{d_i} = \mathcal {M}$ and $\lim _{i\rightarrow \infty } \mathcal {N}^{d_i} = \mathcal {N}$. Using this and (25) we find

$$\begin{aligned} \mathcal {N} \circ \mathcal {M}&= \lim _{i \rightarrow \infty } \mathcal {N}^{d_i} \circ \mathcal {M}^{d_i} \\&= \lim _{i \rightarrow \infty } \ \bigl ( \overline{\mathcal {M}^{d_i}} \otimes \overline{\mathcal {N}^{d_i}} \bigr ) \circ \mathcal {D}^{d_i} \\&= \bigl ( \lim _{i \rightarrow \infty } \overline{\mathcal {M}^{d_i}} \otimes \overline{\mathcal {N}^{d_i}} \bigr ) \circ \bigl (\lim _{i \rightarrow \infty } \mathcal {D}^{d_i} \bigr ) \\&= \bigl ( \lim _{i \rightarrow \infty } \overline{\mathcal {M}^{d_i}} \otimes \overline{\mathcal {N}^{d_i}} \bigr ) \circ \mathcal {D}. \end{aligned}$$

Finally, Remark 2.8 implies that, for arbitrary states $\zeta _I$ and $\zeta _J$, $\overline{\mathcal {M}^{d_i}} = \textrm{tr}_H \circ \mathcal {M}^{d_i} \circ \zeta _J$ and $\overline{\mathcal {N}^{d_i}} = \mathcal {N}^{d_i} \circ \zeta _I$. Hence,

$$\begin{aligned}&\lim _{i \rightarrow \infty } \overline{\mathcal {M}^{d_i}} \otimes \overline{\mathcal {N}^{d_i}}\\&\quad = \lim _{i \rightarrow \infty } \textrm{tr}_{H} \circ \mathcal {M}^{d_i} \circ \zeta _J \otimes \mathcal {N}^{d_i} \circ \zeta _I \\&\quad = \lim _{i \rightarrow \infty } \bigl ( \Pi _A^{d_i} + \zeta _A \circ \textrm{tr}_A \circ (\mathcal {I}_A - \Pi _A^{d_i})\bigr ) \circ \textrm{tr}_H \circ \mathcal {M} \circ \zeta _J \otimes \Pi _B^{d_i} \circ \mathcal {N} \circ \zeta _I \\&\quad = \lim _{i \rightarrow \infty } \Bigl ( \Pi _A^{d_i} \otimes \Pi _B^{d_i} + \zeta _A \circ \textrm{tr}_A \circ (\mathcal {I}_A - \Pi _A^{d_i}) \otimes \Pi _B^{d_i} \Bigr ) \circ \bigl ( \textrm{tr}_H \circ \mathcal {M} \circ \zeta _J \otimes \mathcal {N} \circ \zeta _I \bigr ) \\&\quad = \underbrace{ \lim _{i \rightarrow \infty } \bigl (\Pi _A^{d_i} \otimes \Pi _B^{d_i} \bigr ) \circ \bigl ( \textrm{tr}_H \circ \mathcal {M} \circ \zeta _J \otimes \mathcal {N} \circ \zeta _I \bigr )}_{=\textrm{tr}_H \circ \mathcal {M} \circ \zeta _J \otimes \mathcal {N} \circ \zeta _I} \\&\qquad + \zeta _A \circ \textrm{tr}_A \circ \underbrace{ \lim _{i \rightarrow \infty } \bigl ( (\mathcal {I}_A - \Pi _A^{d_i}) \otimes \Pi _B^{d_i} \bigr ) \circ \bigl (\textrm{tr}_H \circ \mathcal {M} \circ \zeta _J \otimes \mathcal {N} \circ \zeta _I \bigr )}_{= 0}\\&\quad = \textrm{tr}_{H} \circ \mathcal {M} \circ \zeta _J \otimes \mathcal {N} \circ \zeta _I \\&\quad = \overline{\mathcal {M}} \otimes \overline{\mathcal {N}}. \end{aligned}$$

Combining this with the equality above, we obtain (4).

As a preparation for our converse statement, Theorem 3.6, we first establish some general properties of the doubling map $\mathcal {D}$ that comes out of Theorem 3.1.

### Remark 3.5

The CPTP map $\mathcal {D}:K\rightarrow K'\otimes K''$ in Theorem 3.1 fulfils

(i) $${{\,\textrm{tr}\,}}_{\tilde{K}'}\circ \mathcal {D}_{K''\rightarrow \tilde{K}'\tilde{K}''}\circ \mathcal {D}_{K''\rightarrow K' K''}=\mathcal {D}_{K\rightarrow K' \tilde{K}''}$$ and
(ii) ${{\,\textrm{tr}\,}}_{K'}\circ \mathcal {D}_{K\rightarrow K' K''}$ is unital.

(Note that all *K*’s are isomorphic spaces, but we use the notation above to distinguish them to keep track of where the different maps go.)

To prove Property (i), we show how the maps on each side of the equality act on a general basis element $(|a \rangle _{K_A^z}\otimes |b \rangle _{K_B^z})(\langle \bar{a} |_{K_A^{\bar{z}}} \otimes \langle \bar{b} |_{K_B^{\bar{z}}})$ of the space of operators on *K*. For the right-hand side, applying $\mathcal {D}_{K\rightarrow K' \tilde{K}''}$ as defined in (16) and (17) yields26where the colours indicate which parts combined yield a $\delta$-function on the respective labels. The left-hand side of (i) applied to the same bases element yields

$$\begin{aligned}&{{\,\textrm{tr}\,}}_{\tilde{K}'}\circ \mathcal {D}_{K''\rightarrow \tilde{K}'\tilde{K}''}\circ \mathcal {D}_{K''\rightarrow K' K''}\big ((|a \rangle _{K_A^z}\otimes |b \rangle _{K_B^z})(\langle \bar{a} |_{K_A^{\bar{z}}} \otimes \langle \bar{b} |_{K_B^{\bar{z}}})\big )\\&\hspace{20pt}={{\,\textrm{tr}\,}}_{\tilde{K}'}\circ \mathcal {D}_{K''\rightarrow \tilde{K}'\tilde{K}''}\Big (\underbrace{|a \rangle \!\langle \bar{a} |_{K_A'^z}\otimes \,\overline{\textrm{id}}_{K_B'^z}}_{=:\rho _{K'}}\otimes \underbrace{\,\overline{\textrm{id}}_{K_A''^z}}_{=1/\dim (K_A^z)\sum _{\hat{a}}|\hat{a} \rangle \!\langle \hat{a} |_{K_A''^z}}\otimes |b \rangle \!\langle \bar{b} |_{K_B''^z}\Big )\cdot \delta _{z\bar{z}}\\&\hspace{20pt}={{\,\textrm{tr}\,}}_{\tilde{K}'}\Big ({\textstyle \frac{1}{\dim (K_A^z)}} \sum _{\hat{a}}\mathcal {D}_{K''\rightarrow \tilde{K}'\tilde{K}''}\big (|\hat{a} \rangle \!\langle \hat{a} |_{K_A''^z}\otimes |b \rangle \!\langle \bar{b} |_{K_B''^z}\big )\Big )\otimes \rho _{K'}\cdot \delta _{z\bar{z}}\\&\hspace{20pt}={{\,\textrm{tr}\,}}_{\tilde{K}'}\Big (\underbrace{{\textstyle \frac{1}{\dim (K_A^z)}}\sum _{\hat{a}}|\hat{a} \rangle \!\langle \hat{a} |_{\tilde{K}_A'^z}}_{=\,\overline{\textrm{id}}_{\tilde{K}_A'^z}}\otimes |b \rangle \!\langle \bar{b} |_{\tilde{K}_B''^z}\otimes \,\overline{\textrm{id}}_{\tilde{K}_B'^z}\otimes \,\overline{\textrm{id}}_{\tilde{K}_A''^z}\Big )\otimes \rho _{K'}\cdot \delta _{z\bar{z}}\\&\hspace{20pt}=|a \rangle \!\langle \bar{a} |_{K_A'^z}\otimes \,\overline{\textrm{id}}_{K_B'^z}\otimes \,\overline{\textrm{id}}_{\tilde{K}_A''^z}\otimes |b \rangle \!\langle \bar{b} |_{\tilde{K}_B''^z}\cdot \delta _{z\bar{z}}, \end{aligned}$$

which is equal to (26). Since this is true for any basis element of the space of operators on *K*, the maps are equal, proving (i). Property (ii) can be shown by a direct calculation, using that $\textrm{id}_K=\sum _{z,a,b}|a \rangle \!\langle a |_{K_A^z}\otimes |b \rangle \!\langle b |_{K_B^z}$:

$$\begin{aligned} {{\,\textrm{tr}\,}}_{K'}\circ \mathcal {D}_{K\rightarrow K' K''}(\textrm{id}_K)&={{\,\textrm{tr}\,}}_{K'}\Big (\sum _{a,b,z}\mathcal {D}_{K\rightarrow K' K''}\big (|a \rangle \!\langle a |_{K_A^z}\otimes |b \rangle \!\langle b |_{K_B^z}\big )\Big )\\&={{\,\textrm{tr}\,}}_{K'}\Big (\sum _{a,b,z}|a \rangle \!\langle a |_{K_A'^z}\otimes \,\overline{\textrm{id}}_{K_B'^z}\otimes \,\overline{\textrm{id}}_{K_A''^z}\otimes |b \rangle \!\langle b |_{K_B''^z}\Big )\\&=\sum _z\underbrace{{{\,\textrm{tr}\,}}_{K'}(\textrm{id}_{K_A'^z}\otimes \,\overline{\textrm{id}}_{K_B'^z}}_{=\dim (K_A'^z)})\,\overline{\textrm{id}}_{K_A''^z}\otimes \textrm{id}_{K_B''^z}\\&=\sum _z\textrm{id}_{K_A''^z}\otimes \textrm{id}_{K_B''^z}\\&=\textrm{id}_{K''}. \end{aligned}$$

We are now ready to state and prove the converse to Theorem 3.1. Identifying $\mathcal {A}$ and $\mathcal {B}$ with $\overline{\mathcal {M}}$ and $\overline{\mathcal {N}}$, respectively, it implies that the Conditions (i), (ii), and (iii) are necessary.

### Theorem 3.6

(Converse statement to Theorem 3.1). Let $\mathcal {E}:I\otimes K\otimes J\rightarrow A\otimes B$ be a CP map such that $\mathcal {E}=(\mathcal {A}\otimes \mathcal {B})\circ \mathcal {D}$ for a CPTP map $\mathcal {A}:I\otimes K\rightarrow A$ and CP maps $\mathcal {B}:K\otimes J\rightarrow B$ and $\mathcal {D}:K\rightarrow K\otimes K$, where the latter fulfils Properties (i) and (ii) in Remark 3.5.^6^

Then there exist CP maps $\mathcal {M}:I\otimes K\otimes J\rightarrow A\otimes I\otimes K\otimes J$ and $\mathcal {N}:I\otimes K\otimes J\rightarrow B$ such that $\mathcal {E}=\mathcal {N}\circ \mathcal {M}$ and $\mathcal {M}$ and $\mathcal {N}$ fulfil Conditions (i), (ii), and (iii) in Theorem 3.1.

**Fig. 4:**
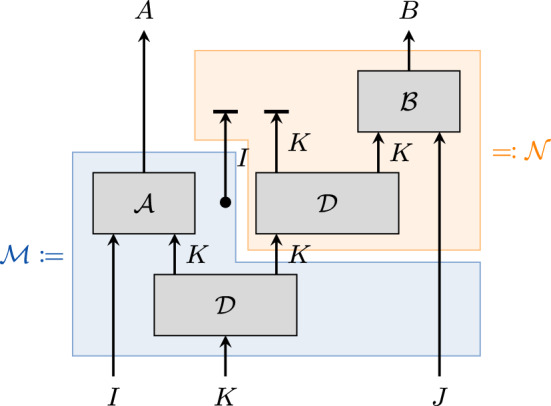
Visualisation of the map $\mathbf {\mathcal {E}}$ **occurring in Theorem** 3.6. The diagram shows the components of the map $\mathcal {E}$ as given in (27). The blue and orange boxes define the maps $\mathcal {M}$ and $\mathcal {N}$, respectively

### Proof

First, note that we can insert a map of the form ${{\,\textrm{tr}\,}}_I\circ \,\overline{\textrm{id}}_{I}$ into $(\mathcal {A}\otimes \mathcal {B})\circ \mathcal {D}$ without changing the map. Together with Property (i) in Remark 3.5 this allows us to write (see Fig. 4)

27 $$\begin{aligned} \mathcal {E}=(\mathcal {A}\otimes \mathcal {B})\circ \mathcal {D}&=(\mathcal {A}\otimes \mathcal {B})\circ {{\,\textrm{tr}\,}}_K\circ \mathcal {D}\circ \mathcal {D}\nonumber \\&=\mathcal {A}\circ \mathcal {B}\circ {{\,\textrm{tr}\,}}_K\circ \mathcal {D}\circ {{\,\textrm{tr}\,}}_I\circ \,\overline{\textrm{id}}_{I}\circ \mathcal {D}\nonumber \\&=\underbrace{\big ({{\,\textrm{tr}\,}}_K\circ \mathcal {B}\circ \mathcal {D}\circ {{\,\textrm{tr}\,}}_I\big )}_{=:\mathcal {N}}\circ \underbrace{\big (\,\overline{\textrm{id}}_{I}\circ \mathcal {A}\circ \mathcal {D}\big )}_{=:\mathcal {M}}. \end{aligned}$$

Note that $\mathcal {M}$ acts on *J* as the identity. We can then show that $\mathcal {M}$ and $\mathcal {N}$ fulfil Conditions (i), (ii), and (iii) in Theorem 3.1:

(i) $${{\,\textrm{tr}\,}}_A\circ \mathcal {N}\circ \mathcal {M} ={{\,\textrm{tr}\,}}_A\circ \big (\mathcal {A}\otimes \mathcal {B}\big )\circ \mathcal {D} ={{\,\textrm{tr}\,}}_K\circ \mathcal {B}\circ \mathcal {D}\circ {{\,\textrm{tr}\,}}_I =\mathcal {N}$$.
(ii) Property (ii) in Remark 3.5 says that ${{\,\textrm{tr}\,}}_K\circ \mathcal {D}$ is unital. Hence,
  $$\begin{aligned} {{\,\textrm{tr}\,}}_A\circ \mathcal {M}(\textrm{id}_{IKJ})&={{\,\textrm{tr}\,}}_A\circ \,\overline{\textrm{id}}_{I}\circ \mathcal {A}\circ \mathcal {D}(\textrm{id}_{IKJ})\\&=\,\overline{\textrm{id}}_{I}\circ \underbrace{{{\,\textrm{tr}\,}}_I(\textrm{id}_I)}_{=\dim (I)}\otimes \underbrace{{{\,\textrm{tr}\,}}_K\circ \mathcal {D}(\textrm{id}_K)}_{\textrm{id}_K}\otimes \textrm{id}_J\\&=\textrm{id}_{IKJ}, \end{aligned}$$
  thus ${{\,\textrm{tr}\,}}_A\circ \mathcal {M}$ is unital.
(iii) From the definition of $\mathcal {M}$ and $\mathcal {N}$ it directly follows that $\textrm{tr}_H \circ \mathcal {M}$ and $\mathcal {N}$ are independent of *J* and *I*, respectively. $\square$

### Remark 3.7

It has been shown in [9] that, if a map is unitary and satisfies a non-signalling condition analogous to Condition (iii) of Theorem 3.1, then this map has a structure similar to the right-hand side of (4). Note that the unitarity assumption is crucial for this result: the PR box [8, 10, 11] does not admit such a structure, although it satisfies the non-signalling condition. In contrast, Theorem 3.1 is valid for general (not necessarily unitary) CP maps, but instead requires the additional Conditions (i) and (ii) (which are also necessary; see Theorem 3.6).

## Implications

**Fig. 5:**
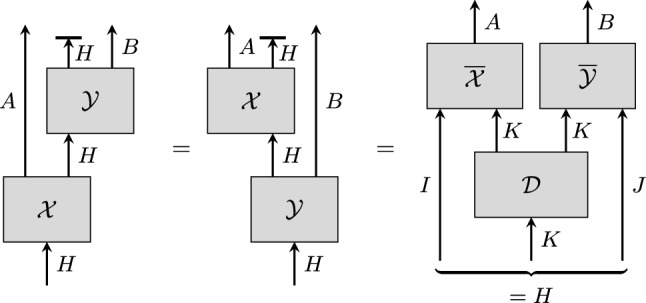
Visualisation of Corollary 4.1. Condition (i) holds if and only if $\mathcal {X}$ and $\mathcal {Y}$ commute, in the sense that the two circuit diagrams on the left have the same input-output behaviour. Provided that the other conditions are also satisfied, the corollary implies that the circuit diagram shown to the right, where $\overline{\mathcal {X}}$ and $\overline{\mathcal {Y}}$ act on two separate copies of *K*, also has the same input-output behaviour. The diagram thus captures the idea that commuting maps factorise

Having established Theorem 3.1, we can give an answer to the question posed in the introduction, generalising Tsirelson’s result [15] to the “fully quantum” case. We state this answer as Corollary 4.1. Figure 5 illustrates the main assumption of the corollary—a commutation relation between the maps ${\mathcal {X}}$ and ${\mathcal {Y}}$ described as Condition (i)—as well as the conclusion, which is that the concatenation of these two maps factorises; see (28).

The special case of Tsirelson’s result, which we discuss later as Corollary 4.2, refers to families of measurement operators $\{X_{i,\alpha }\}$ and $\{Y_{j,\beta }\}$ instead of CP maps ${\mathcal {X}}$ and ${\mathcal {Y}}$. Hence, Condition (i) of Corollary 4.1 can be understood as a quantum generalisation of Tsirelson’s assumption that the families of measurement operators $\{X_{i,\alpha }\}$ and $\{Y_{j,\beta }\}$ commute; see (29). Note that the measurement operators satisfy the property $\smash {\sum _\alpha } X_{i,\alpha }=\textrm{id}_{K}$ and $\smash {\sum _\beta } Y_{j,\beta }=\textrm{id}_{K}$. In Corollary 4.1, this property generalises to a unitality assumption, phrased as Condition (ii).^7^ This assumption is necessary; if we drop it without replacement, the statement is false, even when one restricts it to the purely classical case. This can be seen by choosing the maps ${\mathcal {X}}$ and ${\mathcal {Y}}$ such that $\textrm{tr}_H\circ {\mathcal {Y}}\circ {\mathcal {X}}$ implements the PR box [8, 10, 11]. It is known that the PR box does not factorise, for this would violate the Tsirelson bound [13].^8^ We refer to Appendix B for more details.

Furthermore, the statement of Theorem 3.1 can be generalised to a family consisting of more than two maps which fulfil assumptions similar to Conditions  (i)–(iii) in Theorem 3.1. We present this statement as Corollary 4.5.

### Corollary 4.1

Let ${\mathcal {X}}:H\rightarrow H\otimes A$ and ${\mathcal {Y}}:H\rightarrow H\otimes B$ be CPTP maps, where $H= I\otimes K\otimes J$ is finite-dimensional, such that

(i) $$\textrm{tr}_H\circ {\mathcal {Y}}\circ {\mathcal {X}}=\textrm{tr}_H\circ {\mathcal {X}}\circ {\mathcal {Y}}$$
(ii) either $\textrm{tr}_A\circ {\mathcal {X}}$ or $\textrm{tr}_B\circ {\mathcal {Y}}$ is unital
(iii) $\textrm{tr}_H\circ {\mathcal {X}}$ is independent of *J* and $\textrm{tr}_H\circ {\mathcal {Y}}$ is independent of *I*.

Then there exists a CPTP map $\mathcal {D}:K\rightarrow K\otimes K$ such that

28 $$\begin{aligned} \textrm{tr}_H\circ {\mathcal {Y}}\circ {\mathcal {X}}=\big (\overline{\mathcal {X}}\otimes \overline{\mathcal {Y}}\big )\circ \mathcal {D}, \end{aligned}$$

where $$\overline{\mathcal {X}}\circ {{\,\textrm{tr}\,}}_J={{\,\textrm{tr}\,}}_H\circ \mathcal {X}$$, $$\overline{\mathcal {Y}}\circ {{\,\textrm{tr}\,}}_I={{\,\textrm{tr}\,}}_H\circ \mathcal {Y}$$.

### Proof

Without loss of generalisation, we assume that $\textrm{tr}_A\circ {\mathcal {X}}$ is unital. If instead $\textrm{tr}_B\circ {\mathcal {Y}}$ is unital the same proof works by exchanging the roles of ${\mathcal {X}}$ and ${\mathcal {Y}}$. To apply Theorem 3.1, we set $\mathcal {M}:={\mathcal {X}}$ and $\mathcal {N}:=\textrm{tr}_H\circ {\mathcal {Y}}$, thus $\mathcal {N}\circ \mathcal {M}=\textrm{tr}_H\circ {\mathcal {Y}}\circ {\mathcal {X}}$. Because ${\mathcal {X}}$ and ${\mathcal {Y}}$ commute under the trace over *H*, it follows that

$$\begin{aligned} \textrm{tr}_A\circ \mathcal {N}\circ \mathcal {M}=\textrm{tr}_{AH}\circ {\mathcal {Y}}\circ {\mathcal {X}}=\textrm{tr}_{AH}\circ {\mathcal {X}}\circ {\mathcal {Y}} =\textrm{tr}_H\circ {\mathcal {Y}} =\mathcal {N}, \end{aligned}$$

hence Condition (i) in Theorem 3.1 is fulfilled. Conditions (ii) and (iii) are directly fulfilled by the definition of the maps ${\mathcal {X}},{\mathcal {Y}}$. Hence, we can apply Theorem 3.1 and the statement of the corollary directly follows. $\square$

If we specialise Corollary 4.1 to the case where the inputs *I*, *J* and the outputs *A*, *B* are classical, we retrieve the statement of [15] as described above. In fact, one may more generally consider a classical version of Theorem 3.1 instead of Corollary 4.1. This yields another generalisation of Tsirelson’s result, stated in Remark 4.4, which may be of independent interest.

### Corollary 4.2

Let $\{X_{i,\alpha }\}$ and $\{Y_{j,\beta }\}$ be finite families of positive operators on a finite-dimensional Hilbert space *K* such that

29 $$\begin{aligned} {[}X_{i,\alpha },Y_{j,\beta }]=0 \quad \forall \, i,j,\alpha ,\beta , \end{aligned}$$

and $\sum _\alpha X_{i,\alpha }=\textrm{id}_{K}$ and $\sum _\beta Y_{j,\beta }=\textrm{id}_{K}$ for all *i*, *j*. Then there exists another finite-dimensional Hilbert space $\overline{K}$ with decomposition $\overline{K}=K_A\otimes K_B$ and an isometry $V: K\rightarrow \overline{K}$ such that

30 $$\begin{aligned} X_{i,\alpha }= V^* \bigl (A_{i,\alpha }\otimes \textrm{id}_{K_B}\bigr ) V ,\hspace{10pt} Y_{j,\beta }= V^* \bigl (\textrm{id}_{K_A}\otimes B_{j,\beta } \bigr ) V, \end{aligned}$$

where $A_{i,\alpha }$ and $B_{j,\beta }$ are operators on $K_A$ and $K_B$, respectively.

### Proof

The first step in the proof is to apply Corollary 4.1 to the setting described above, therefore we have to identify the corresponding Hilbert spaces and maps and show that they fulfil the conditions of the theorem. Let

$$\begin{aligned} I&:=\textrm{span}\{|i \rangle \}_i\\ J&:=\textrm{span}\{|j \rangle \}_j\\ H&:=I\otimes K\otimes J\\ A&:=\textrm{span}\{|\alpha \rangle \}_{\alpha }\\ B&:=\textrm{span}\{|\beta \rangle \}_{\beta }, \end{aligned}$$

where $\{|i \rangle \}_i,\{|j \rangle \}_j,\{|\alpha \rangle \}_{\alpha }$, and $\{|\beta \rangle \}_{\beta }$ are orthonormal families of vectors. Define the maps ${\mathcal {X}}:H\rightarrow A\otimes H$ and ${\mathcal {Y}}:H\rightarrow B\otimes H$ via

31 $$\begin{aligned} \begin{aligned} {\mathcal {X}}&: W_{H}\mapsto \sum _{i,\alpha }\left( |i \rangle \!\langle i |_{I}\otimes \sqrt{X_{i,\alpha }}\otimes \textrm{id}_{J}\right) W_{H}\left( |i \rangle \!\langle i |_{I}\otimes \sqrt{X_{i,\alpha }}\otimes \textrm{id}_{J}\right) \otimes |\alpha \rangle \!\langle \alpha |_A\\ {\mathcal {Y}}&: W_{H}\mapsto \sum _{j,\beta }\left( \textrm{id}_{I}\otimes \sqrt{Y_{j,\beta }}\otimes |j \rangle \!\langle j |_{J}\right) W_{H}\left( \textrm{id}_{I}\otimes \sqrt{Y_{j,\beta }}\otimes |j \rangle \!\langle j |_{J}\right) \otimes |\beta \rangle \!\langle \beta |_B. \end{aligned} \end{aligned}$$

These maps are indeed CPTP maps, which can be shown by identifying their respective Kraus operators. We demonstrate this here for the map ${\mathcal {X}}$: Let ${\mathcal {X}}(W_H)=\sum _{i,\alpha } E_{i,\alpha }W_H E_{i,\alpha }^*$, where $E_{i,\alpha }:H\rightarrow A\otimes H$ are the Kraus operators of ${\mathcal {X}}$ given by

$$\begin{aligned} E_{i,\alpha }:=|i \rangle \!\langle i |_{I}\otimes \sqrt{X_{i,\alpha }}\otimes \textrm{id}_{J}\otimes |\alpha \rangle _A. \end{aligned}$$

The set $\{E_{i,\alpha }\}$ forms indeed a valid set of Kraus operators of a TP map:

$$\begin{aligned} \sum _{i,\alpha }E_{i,\alpha }^*E_{i,\alpha }&=\sum _{i,\alpha }\big (|i \rangle \!\langle i |_I\otimes \sqrt{X_{i,\alpha }}\otimes \textrm{id}_{J}\otimes \langle \alpha |_A\big )\big (|i \rangle \!\langle i |_I\otimes \sqrt{X_{i,\alpha }}\otimes \textrm{id}_{J}\otimes |\alpha \rangle _A\big )\\&=\sum _{i,\alpha }|i \rangle \!\langle i |_I\otimes {X_{i,\alpha }}\otimes \textrm{id}_{J}\otimes \underbrace{\langle \alpha |\alpha \rangle _A}_{=1}\\&=\sum _{i}|i \rangle \!\langle i |_I\otimes \sum _{\alpha }{X_{i,\alpha }}\otimes \textrm{id}_{J}\\&=\sum _i |i \rangle \!\langle i |_I\otimes \textrm{id}_K\otimes \textrm{id}_{J}\\&=\textrm{id}_{H}. \end{aligned}$$

The same calculation can be done for ${\mathcal {Y}}$. Hence, the maps are indeed CPTP maps.

The definition of the maps in (31) directly allows us to show that the conditions in Corollary 4.1 are fulfilled:

1. The maps commute: From $\left[ X_{i,\alpha },Y_{j,\beta }\right] =0$ it follows that $\left[ \sqrt{X_{i,\alpha }},\sqrt{Y_{j,\beta }}\right] =0$ for all $i,j,\alpha ,\beta$. Hence,
  $$\begin{aligned}&\textrm{tr}_H\circ {\mathcal {Y}}\circ {\mathcal {X}}(W_H)\\&\quad =\textrm{tr}_H\Big (\sum _{i,\alpha ,j,\beta }\big (|i \rangle \!\langle i |_I\otimes \sqrt{Y_{j,\beta }}\sqrt{X_{i,\alpha }}\otimes |j \rangle \!\langle j |_J\big )W_H\big (|i \rangle \!\langle i |_I\otimes \sqrt{Y_{j,\beta }}\sqrt{X_{i,\alpha }}\otimes |j \rangle \!\langle j |_J\big )\\&\hspace{70pt}\otimes |\alpha \rangle \!\langle \alpha |_A\otimes |\beta \rangle \!\langle \beta |_B\Big )\\&\quad =\textrm{tr}_H\Big (\sum _{i,\alpha ,j,\beta }\big (|i \rangle \!\langle i |_I\otimes \sqrt{X_{i,\alpha }}\sqrt{Y_{j,\beta }}\otimes |j \rangle \!\langle j |_J\big )W_H\big (|i \rangle \!\langle i |_I\otimes \sqrt{X_{i,\alpha }}\sqrt{Y_{j,\beta }}\otimes |j \rangle \!\langle j |_J\big )\\&\hspace{70pt}\otimes |\alpha \rangle \!\langle \alpha |_A\otimes |\beta \rangle \!\langle \beta |_B\Big )\\&\quad =\textrm{tr}_H\circ {\mathcal {X}}\circ {\mathcal {Y}}(W_H). \end{aligned}$$
2. $$\textrm{tr}_A\circ {\mathcal {X}}$$ is unital:
  $$\begin{aligned}&\textrm{tr}_A\circ {\mathcal {X}}(\textrm{id}_H)\\&\quad =\textrm{tr}_A\left( \sum _{i,\alpha }\left( |i \rangle \!\langle i |_I\otimes \sqrt{X_{i,\alpha }}\otimes \textrm{id}_{J}\right) \textrm{id}_H\left( |i \rangle \!\langle i |_I\otimes \sqrt{X_{i,\alpha }}\otimes \textrm{id}_{J}\right) \otimes |\alpha \rangle \!\langle \alpha |_A\right) \\&\quad =\sum _{i,\alpha }|i \rangle \!\langle i |_I\otimes X_{i,\alpha }\otimes \textrm{id}_{J}\\&\quad =\textrm{id}_H. \end{aligned}$$
3. $\textrm{tr}_H\circ {\mathcal {X}}$ is independent of *J*, i.e., there exists a map $\overline{\mathcal {X}}:I\otimes K\rightarrow A$ such that $\textrm{tr}_H\circ {\mathcal {X}}=\overline{\mathcal {X}}\circ \textrm{tr}_{J}$:
  $$\begin{aligned}&\textrm{tr}_H\circ {\mathcal {X}}(W_H)\\&\quad =\textrm{tr}_{H}\sum _{i,\alpha }\left( |i \rangle \!\langle i |_I\otimes \sqrt{X_{i,\alpha }}\otimes \textrm{id}_{J}\right) W_H\left( |i \rangle \!\langle i |_I\otimes \sqrt{X_{i,\alpha }}\otimes \textrm{id}_{J}\right) \otimes |\alpha \rangle \!\langle \alpha |_A\\&\quad =\textrm{tr}_{IK}\sum _{i,\alpha }\left( |i \rangle \!\langle i |_I\otimes \sqrt{X_{i,\alpha }}\right) \textrm{tr}_{J}(W_H)\left( |i \rangle \!\langle i |_I\otimes \sqrt{X_{i,\alpha }}\right) \otimes |\alpha \rangle \!\langle \alpha |_A\\&\quad =\overline{\mathcal {X}}\circ \textrm{tr}_{J}(W_H) \end{aligned}$$
  with $\overline{\mathcal {X}}(W_{IK}):=\textrm{tr}_{IK}\Big (\sum _{i,\alpha }\left( |i \rangle \!\langle i |_I\otimes \sqrt{X_{i,\alpha }}\right) W_{IK}\left( |i \rangle \!\langle i |_I\otimes \sqrt{X_{i,\alpha }}\right) \otimes |\alpha \rangle \!\langle \alpha |_A\Big )$. The statement that $\textrm{tr}_H\circ {\mathcal {Y}}$ is independent of *I* can be shown analogously.

Hence, all conditions in Corollary 4.1 are fulfilled and it follows that there exists a CPTP map $\mathcal {D}:K\rightarrow K\otimes K$ such that

$$\begin{aligned} \textrm{tr}_H\circ {\mathcal {Y}}\circ {\mathcal {X}}=(\overline{\mathcal {X}}\otimes \overline{\mathcal {Y}})\circ \mathcal {D}, \end{aligned}$$

where $$\overline{\mathcal {X}}\circ {{\,\textrm{tr}\,}}_J={{\,\textrm{tr}\,}}_H\circ \mathcal {X}$$, $$\overline{\mathcal {Y}}\circ {{\,\textrm{tr}\,}}_I={{\,\textrm{tr}\,}}_H\circ \mathcal {Y}$$.

Next, we need to find the isometries that map the operators $X_{i,\alpha },Y_{j,\beta }$ on *K* to the product Hilbert space $K\otimes K$ and the corresponding isometric operators. For this purpose, we first define CPTP maps $\overline{\mathcal {X}}_i,\overline{\mathcal {Y}}_j$ via

32 $$\begin{aligned} \overline{\mathcal {X}}_i:&W_{K}\mapsto \sum _{\alpha }|\alpha \rangle \!\langle \alpha |_A\ \overline{\mathcal {X}}\big (|i \rangle \!\langle i |_{I}\otimes W_{K}\big )|\alpha \rangle \!\langle \alpha |_A \end{aligned}$$

33 $$\begin{aligned} \overline{\mathcal {Y}}_j:&W_{K}\mapsto \sum _{\beta }|\beta \rangle \!\langle \beta |_B\ \overline{\mathcal {Y}}\big (|j \rangle \!\langle j |_{J}\otimes W_{K}\big )|\beta \rangle \!\langle \beta |_B \end{aligned}$$

and show that

34 $$\begin{aligned} \textrm{tr}\big (X_{i,\alpha }Y_{j,\beta }\rho _K\big )=\big (\overline{\mathcal {X}}_{i,\alpha }\otimes \overline{\mathcal {Y}}_{j,\beta }\big )\circ \mathcal {D}(\rho _K), \end{aligned}$$

where $\overline{\mathcal {X}}_{i,\alpha }:=\langle \alpha |\overline{\mathcal {X}}_i|\alpha \rangle$, $\overline{\mathcal {Y}}_{j,\beta }:=\langle \beta |\overline{\mathcal {Y}}_j|\beta \rangle$. The proof goes as follows: For any state $\rho _K$ on *K* and all *i*, *j*, it follows from (31) and commutativity that

$$\begin{aligned}&\sum _{\alpha ,\beta }\textrm{tr}_K\big (X_{i,\alpha }Y_{j,\beta }\rho _K\big )\otimes |\alpha \rangle \!\langle \alpha |_A\otimes |\beta \rangle \!\langle \beta |_B = \textrm{tr}_H \circ {\mathcal {Y}}\circ {\mathcal {X}}\big (|i \rangle \!\langle i |_{I}\otimes \rho _K\otimes |j \rangle \!\langle j |_{J}\big ) \\&\qquad = (\overline{\mathcal {X}}\otimes \overline{\mathcal {Y}})\circ \mathcal {D} \big (|i \rangle \!\langle i |_{I}\otimes \rho _K\otimes |j \rangle \!\langle j |_{J}\big ) \\&\qquad = \big (\overline{\mathcal {X}}\otimes \overline{\mathcal {Y}}\big )\, \big (|i \rangle \!\langle i |_{I}\otimes \mathcal {D}(\rho _K)\otimes |j \rangle \!\langle j |_{J}\big ). \end{aligned}$$

We may now apply the map $W_{A B} \mapsto \sum _{\tilde{\alpha }, \tilde{\beta }} |\tilde{\alpha } \rangle \!\langle \tilde{\alpha } | \otimes |\tilde{\beta } \rangle \!\langle \tilde{\beta } | W_{A B} |\tilde{\alpha } \rangle \!\langle \tilde{\alpha } | \otimes |\tilde{\beta } \rangle \!\langle \tilde{\beta } |$ to the first and the last expression in this equality. Since this map acts like an identity on the first, we obtain

$$\begin{aligned} \sum _{\tilde{\alpha },\tilde{\beta }}&\textrm{tr}_K\big (X_{i,\tilde{\alpha }}Y_{j,\tilde{\beta }}\rho _K\big )\otimes |\tilde{\alpha } \rangle \!\langle \tilde{\alpha } |_A\otimes |\tilde{\beta } \rangle \!\langle \tilde{\beta } |_B\\&=\sum _{\tilde{\alpha },\tilde{\beta }}|\tilde{\alpha } \rangle \!\langle \tilde{\alpha } |_A\otimes |\tilde{\beta } \rangle \!\langle \tilde{\beta } |_B\Big [\big (\overline{\mathcal {X}}\otimes \overline{\mathcal {Y}}\big )\, \big (|i \rangle \!\langle i |_{I}\otimes \mathcal {D}(\rho _K)\otimes |j \rangle \!\langle j |_{J}\big )\Big ]|\tilde{\alpha } \rangle \!\langle \tilde{\alpha } |_A\otimes |\tilde{\beta } \rangle \!\langle \tilde{\beta } |_B\\&=(\overline{\mathcal {X}}_i\otimes \overline{\mathcal {Y}}_j)\circ \mathcal {D}(\rho _K), \end{aligned}$$

where we have used the definitions (32) and (33). Sandwiching this equality with $|\alpha \rangle _A\otimes |\beta \rangle _B$ yields (34), which we wanted to show.

Next, note that $\overline{\mathcal {X}}_{i,\alpha }$ and $\overline{\mathcal {Y}}_{j,\beta }$ are CP maps from *K* to a one-dimensional system. According to Lemma 2.15 there exist Hermitian operators $\overline{X}_{i,\alpha }$ and $\overline{Y}_{j,\beta }$ such that

35 $$\begin{aligned} \begin{aligned} \overline{\mathcal {X}}_{i,\alpha }(W_{K})&=\textrm{tr}\left( \overline{X}_{i,\alpha } W_{K}\right) \\ \overline{\mathcal {Y}}_{j,\beta }(W_{K})&=\textrm{tr}\left( \overline{Y}_{j,\beta }W_{K}\right) , \end{aligned} \end{aligned}$$

hence

$$\begin{aligned} \big (\overline{\mathcal {X}}_{i,\alpha }\otimes \overline{\mathcal {Y}}_{j,\beta }\big )(W_{KK})=\textrm{tr}\big ((\overline{X}_{i,\alpha }\otimes \overline{Y}_{j,\beta })W_{KK}\big ). \end{aligned}$$

Thus, (34) can be rewritten as

36 $$\begin{aligned} \textrm{tr}\big (X_{i,\alpha }Y_{j,\beta }\rho _K\big )&=\textrm{tr}\big ((\overline{X}_{i,\alpha }\otimes \overline{Y}_{j,\beta })\circ \mathcal {D}(\rho _K)\big ). \end{aligned}$$

For the next step, we use that according to the Stinespring dilation, there exists an isometric map $\overline{\mathcal {D}}:K\rightarrow K\otimes K\otimes R$ such that ${{\,\textrm{tr}\,}}_R\circ \overline{\mathcal {D}}=\mathcal {D}$, i.e., $\overline{\mathcal {D}}(\rho _K)={V}\rho _K{V}^*$ for some isometry ${V}:K\rightarrow K\otimes K\otimes R$. Hence, (36) can be written as

$$\begin{aligned} \textrm{tr}\big (X_{i,\alpha }Y_{j,\beta }\rho _K\big )&=\textrm{tr}\big ((\overline{X}_{i,\alpha }\otimes \overline{Y}_{j,\beta }\otimes \textrm{id}_R)\circ \overline{\mathcal {D}}(\rho _K)\big )\\&=\textrm{tr}\big ((\overline{X}_{i,\alpha }\otimes \overline{Y}_{j,\beta }\otimes \textrm{id}_R){V}\rho _K{V}^*\big )\\&=\textrm{tr}\big ({V}^*(\overline{X}_{i,\alpha }\otimes \overline{Y}_{j,\beta }\otimes \textrm{id}_R){V}\rho _K\big ). \end{aligned}$$

This is true for any $\rho _K\in K$, hence

37 $$\begin{aligned} X_{i,\alpha }Y_{j,\beta }={V}^*(\overline{X}_{i,\alpha }\otimes \overline{Y}_{j,\beta }\otimes \textrm{id}_R){V}. \end{aligned}$$

Since $\overline{\mathcal {Y}}_j$ is trace-preserving, $\sum _{\beta }\overline{\mathcal {Y}}_{j,\beta }$ is also trace-preserving:

$$\begin{aligned} \textrm{tr}\Big (\sum _{\beta }\overline{\mathcal {Y}}_{j,\beta }(W_K)\Big )=\sum _{\beta }\textrm{tr}\big (\langle \beta |\overline{\mathcal {Y}}_{j}(W_K)|\beta \rangle \big )=\textrm{tr}\big (\overline{\mathcal {Y}}_{j}(W_K)\big )=\textrm{tr}(W_K). \end{aligned}$$

Because this holds for all $W_K$ and all *j*, combining it with (35) yields that the operators $\overline{Y}_{j,\beta }$ fulfil $\sum _{\beta }\overline{Y}_{j,\beta }=\textrm{id}_{K}$ for all *j*. Similarly, we find that $\sum _{\alpha }\overline{X}_{i,\alpha }=\textrm{id}_{K}$ for all *i*. Summing over $\beta$ in (37) then yields

38 $$\begin{aligned} X_{i,\alpha }={V}^*\big (\overline{X}_{i,\alpha }\otimes \textrm{id}_{K}\otimes \textrm{id}_R\big ){V} \end{aligned}$$

and, similarly, summing over $\alpha$ yields

39 $$\begin{aligned} Y_{j,\beta }={V}^*\big (\textrm{id}_{K}\otimes \overline{Y}_{j,\beta }\otimes \textrm{id}_R\big ){V}. \end{aligned}$$

With the identification $K_A\equiv K$, $K_B\equiv K\otimes R$, (38) and (39) say that $X_{i,\alpha }$ and $Y_{j,\beta }$ are isometrically represented as operators on $\overline{K}=K_A\otimes K_B$ that act non-trivially only on $K_A$ and $K_B$, respectively. $\square$

### Remark 4.3

It is actually not necessary in Corollary 4.2 to assume that for all *i*, *j*, $\sum _\alpha X_{i,\alpha }=\textrm{id}_K$ and $\sum _\beta Y_{j,\beta }=\textrm{id}_K$. If this does not hold, we can scale the operators with a constant $\gamma >0$ such that $\sum _{\alpha } X_{i, \alpha } \le \frac{1}{\gamma }\textrm{id}_K$ and add the operator $X_{i,0}:=\textrm{id}_K-\gamma \sum _\alpha X_{i,\alpha }$ (and analogously for *Y*). This operator is also positive and commutes with all operators in $\{Y_{j,\beta }\}$.

### Remark 4.4

As described above, Corollary 4.2 is obtained from Corollary 4.1 by treating *I*, *J*, *A*, and *B* as classical systems. We could apply the same procedure directly to Theorem 3.1. This allows us to derive a stronger version of Corollary 4.2, where (29) is replaced by the weaker condition that the two families of operators $\{X_{i,\alpha }\}$ and $\{Y_{j,\beta }\}$ satisfy

40 $$\begin{aligned} \sum _\alpha \sqrt{X_{i, \alpha }} Y_{j, \beta } \sqrt{X_{i, \alpha }} = Y_{j, \beta } \quad \text { and } \forall \, i, j, \beta \ . \end{aligned}$$

Although the condition merely involves a sum over $\alpha$ rather than a commutation relation for each $\alpha$, it suffices to imply that the operators factorise as in (30).

To prove this, we define, analogously to (31),

$$\begin{aligned} \begin{aligned} \mathcal {M}&: W_{H}\mapsto \sum _{i,\alpha }\left( |i \rangle \!\langle i |_{I}\otimes \sqrt{X_{i,\alpha }}\otimes \textrm{id}_{J}\right) W_{H}\left( |i \rangle \!\langle i |_{I}\otimes \sqrt{X_{i,\alpha }}\otimes \textrm{id}_{J}\right) \otimes |\alpha \rangle \!\langle \alpha |_A,\\ \mathcal {N}&: W_{H}\mapsto \sum _{j,\beta } \textrm{tr}_H \left( \textrm{id}_{I}\otimes \sqrt{Y_{j,\beta }}\otimes |j \rangle \!\langle j |_{J}\right) W_{H}\left( \textrm{id}_{I}\otimes \sqrt{Y_{j,\beta }}\otimes |j \rangle \!\langle j |_{J}\right) \otimes |\beta \rangle \!\langle \beta |_B. \end{aligned} \end{aligned}$$

These CP maps manifestly satisfy Condition (iii) of Theorem 3.1. Furthermore, since $\mathcal {M}$ is identical to ${\mathcal {X}}$ as defined in the proof of Corollary 4.2, we already know that ${{\,\textrm{tr}\,}}_A \circ \mathcal {M}$ is unital and trace-preserving, so Condition (ii) holds. Finally, Condition (i) is equivalent to the requirement that, for all $\rho _K$ and all *i*, *j*,

$$\begin{aligned} \sum _{\beta } {{\,\textrm{tr}\,}}_A \circ \mathcal {N} \circ \mathcal {M}(|i \rangle \!\langle i | \otimes \rho _K \otimes |j \rangle \langle j |) = \sum _{\beta } \mathcal {N}(|i \rangle \!\langle i | \otimes \rho _K \otimes |j \rangle \langle j |). \end{aligned}$$

Inserting the explicit expressions for the maps, the requirement can be rewritten as

$$\begin{aligned} \sum _{\alpha ,\beta } {{\,\textrm{tr}\,}}\big (Y_{j, \beta } \sqrt{X_{i, \alpha }} \rho _K \sqrt{X_{i, \alpha }}\big )\otimes |\beta \rangle \!\langle \beta |_B = \sum _{\beta }{{\,\textrm{tr}\,}}\big (Y_{j, \beta } \rho _K\big )\otimes |\beta \rangle \!\langle \beta |_B \quad \forall \rho _K, i, j. \end{aligned}$$

In particular, the equality must hold individually for each term of the sum over $\beta$. It is thus equivalent to

$$\begin{aligned} \sum _{\alpha } {{\,\textrm{tr}\,}}\big (Y_{j, \beta } \sqrt{X_{i, \alpha }} \rho _K \sqrt{X_{i, \alpha }}\big ) = {{\,\textrm{tr}\,}}\big (Y_{j, \beta } \rho _K\big ) \quad \forall \rho _K, i, j,\beta , \end{aligned}$$

which in turn is equivalent to (40).

### Corollary 4.5

Let $\mathcal {M}_1,\dots ,\mathcal {M}_s$ be CPTP maps such that $\mathcal {M}_i:I_i \otimes K\rightarrow A_i \otimes K$, and ${{\,\textrm{tr}\,}}_{A_i}\circ \,\overline{\textrm{id}}_{I_i}\circ \mathcal {M}_i$ is unital.^9^ If, for all $t\in \{1,\dots ,s-1\}$,

41 $$\begin{aligned} {{\,\textrm{tr}\,}}_{A_1,\dots , A_t}\circ {{\,\textrm{tr}\,}}_K\circ \mathcal {M}_s\circ \dots \circ \mathcal {M}_1={{\,\textrm{tr}\,}}_K\circ \mathcal {M}_s\circ \dots \circ \mathcal {M}_{t+1}\circ {{\,\textrm{tr}\,}}_{I_t}\circ \dots \circ {{\,\textrm{tr}\,}}_{I_1}, \end{aligned}$$

then there exists a CPTP map $\mathcal {D}:K\rightarrow K\otimes K\otimes \dots \otimes K$ such that

42 $$\begin{aligned} {{\,\textrm{tr}\,}}_K\circ \mathcal {M}_s\circ \dots \circ \mathcal {M}_1=\left( \overline{\mathcal {M}}_1\otimes \dots \otimes \overline{\mathcal {M}}_s\right) \circ \mathcal {D}, \end{aligned}$$

where $$\overline{\mathcal {M}}_i={{\,\textrm{tr}\,}}_K\circ \mathcal {M}_i$$.

### Proof

We will prove the statement via iteratively applying Theorem 3.1. First, note that we can always insert a map ${{\,\textrm{tr}\,}}_{I_i}\circ \,\overline{\textrm{id}}_{I_i}$ without changing the left-hand side of (42), for example,

$$\begin{aligned} {{\,\textrm{tr}\,}}_K\circ \mathcal {M}_s\dots \circ \mathcal {M}_1={{\,\textrm{tr}\,}}_K\circ \mathcal {M}_s\dots \circ \mathcal {M}_2\circ {{\,\textrm{tr}\,}}_{I_1}\circ \,\overline{\textrm{id}}_{I_1}\circ \mathcal {M}_1. \end{aligned}$$

Thus, for the first iteration, we choose $\mathcal {M}^{(1)}:=\,\overline{\textrm{id}}_{I_1}\circ \mathcal {M}_1$ and $\mathcal {N}^{(1)}:={{\,\textrm{tr}\,}}_K\circ \mathcal {M}_s\circ \dots \circ \mathcal {M}_2\circ {{\,\textrm{tr}\,}}_{I_1}$, as well as $I^{(1)}:=I_1$ and $J^{(1)}:=I_2\dots I_s$. With these definitions, all conditions in Theorem 3.1 are fulfilled:

(i) From (41), it directly follows that
  $$\begin{aligned} {{\,\textrm{tr}\,}}_{A_1}\circ \mathcal {N}^{(1)}\circ \mathcal {M}^{(1)}&={{\,\textrm{tr}\,}}_{A_1}\circ \left( {{\,\textrm{tr}\,}}_K\circ \mathcal {M}_s\circ \dots \circ \mathcal {M}_2\circ {{\,\textrm{tr}\,}}_{I_1}\right) \circ \left( \,\overline{\textrm{id}}_{I_1}\circ \mathcal {M}_1\right) \\&={{\,\textrm{tr}\,}}_{A_1}\circ {{\,\textrm{tr}\,}}_K\circ \mathcal {M}_s\circ \dots \circ \mathcal {M}_1\\&={{\,\textrm{tr}\,}}_K\circ \mathcal {M}_s\circ \dots \circ \mathcal {M}_2\circ {{\,\textrm{tr}\,}}_{I_1}\\&=\mathcal {N}^{(1)}. \end{aligned}$$
(ii) ${{\,\textrm{tr}\,}}_{A_1}\circ \mathcal {M}^{(1)}={{\,\textrm{tr}\,}}_{A_1}\circ \,\overline{\textrm{id}}_{I_1}\circ \mathcal {M}_1$ is unital by assumption.
(iii) ${{\,\textrm{tr}\,}}_H\circ \mathcal {M}^{(1)}={{\,\textrm{tr}\,}}_H\circ \,\overline{\textrm{id}}_{I_1}\circ \mathcal {M}_1={{\,\textrm{tr}\,}}_K\circ \mathcal {M}_1\circ {{\,\textrm{tr}\,}}_{I_2\dots I_s}$ and $\mathcal {N}^{(1)}={{\,\textrm{tr}\,}}_K\circ \mathcal {M}_s\circ \dots \circ \mathcal {M}_2\circ {{\,\textrm{tr}\,}}_{I_1}$ are obviously independent of $J^{(1)}=I_2\dots I_s$ and $I^{(1)}=I_1$, respectively.

Thus, we can apply Theorem 3.1, which implies the existence of a CPTP map $\mathcal {D}^{(1)}:K\rightarrow K\otimes K$ such that

43 $$\begin{aligned} \mathcal {N}^{(1)}\circ \mathcal {M}^{(1)}=\left( \overline{\mathcal {M}^{(1)}}\otimes \overline{\mathcal {N}^{(1)}}\right) \circ \mathcal {D}^{(1)}, \end{aligned}$$

where $\overline{\mathcal {M}^{(1)}}:={{\,\textrm{tr}\,}}_K\circ \mathcal {M}_1$ and $\overline{\mathcal {N}^{(1)}}:={{\,\textrm{tr}\,}}_K\circ \mathcal {M}_s\circ \dots \mathcal {M}_2$. Thus, (43) translates to

44 $$\begin{aligned} {{\,\textrm{tr}\,}}_K\circ \mathcal {M}_s\circ \dots \circ \mathcal {M}_1=\Bigl (\bigl (\underbrace{{{\,\textrm{tr}\,}}_K\circ \mathcal {M}_1}_{=\overline{\mathcal {M}^{(1)}}}\bigr )\otimes \bigl (\underbrace{{{\,\textrm{tr}\,}}_K\circ \mathcal {M}_s\circ \dots \circ \mathcal {M}_2}_{=\overline{\mathcal {N}^{(1)}}}\bigr )\Bigr ) \circ \mathcal {D}^{(1)}. \end{aligned}$$

Iteration steps 2 to $s-1$ then work similarly: First, we show that (41) implies that for all $t\in \{1,\dots ,s-1\}$,

45 $$\begin{aligned} {{\,\textrm{tr}\,}}_{A_{t}}\circ {{\,\textrm{tr}\,}}_K\circ \mathcal {M}_s\circ \dots \circ \mathcal {M}_{t}={{\,\textrm{tr}\,}}_K\circ \mathcal {M}_s\circ \dots \circ \mathcal {M}_{t+1}\circ {{\,\textrm{tr}\,}}_{I_t}. \end{aligned}$$

This can be derived by applying (41) twice:

$$\begin{aligned} {{\,\textrm{tr}\,}}_{A_t}\circ {{\,\textrm{tr}\,}}_K\circ \mathcal {M}_s\circ \dots \circ \mathcal {M}_{t}\circ {{\,\textrm{tr}\,}}_{I_{t-1}\dots I_1}&={{\,\textrm{tr}\,}}_{A_t} \circ {{\,\textrm{tr}\,}}_{A_{t-1}\dots A_1}\circ {{\,\textrm{tr}\,}}_K\circ \mathcal {M}_s\circ \dots \circ \mathcal {M}_1\\&={{\,\textrm{tr}\,}}_K\circ \mathcal {M}_s\circ \dots \circ \mathcal {M}_{t+1}\circ {{\,\textrm{tr}\,}}_{I_t}\circ {{\,\textrm{tr}\,}}_{I_{t-1}\dots I_1}. \end{aligned}$$

**Fig. 6:**
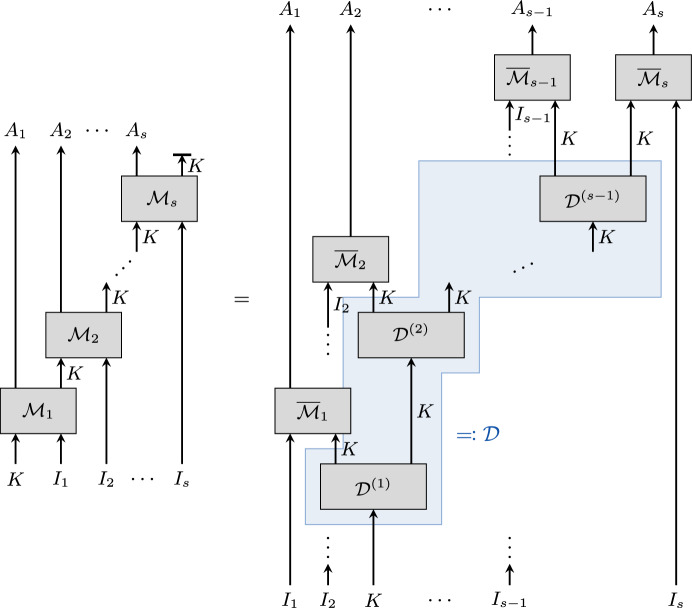
Visualisation of Corollary 4.5. The diagram shows (42), which generalises the statement of Theorem 3.1 to a sequence of *s* maps. Here, $\overline{\mathcal {M}}_i={{\,\textrm{tr}\,}}_K\circ \mathcal {M}_i$ and $\mathcal {D}:=\mathcal {D}^{(s-1)}\circ \dots \circ \mathcal {D}^{(1)}$ (depicted in blue)

We now set $\mathcal {M}^{(t)}:=\,\overline{\textrm{id}}_{I_t}\circ \mathcal {M}_t$ and $\mathcal {N}^{(t)}:={{\,\textrm{tr}\,}}_K\circ \mathcal {M}_s\circ \dots \circ \mathcal {M}_{t+1}\circ {{\,\textrm{tr}\,}}_{I_t}$, and $I^{(t)}:=I_t$, $J^{(t)}:=I_{t+1}\dots I_s$. For this choice, the assumptions of Theorem 3.1 are fulfilled: ${{\,\textrm{tr}\,}}_{A_t}\circ \,\overline{\textrm{id}}_{I_t}\circ \mathcal {M}_t$ is unital by assumption, and it is clear that $\mathcal {M}^{(t)}$ and $\mathcal {N}^{(t)}$ are independent of $I_{t+1}\dots I_s$ and $I_t$, respectively. Furthermore, (45) implies that Condition (i) is fulfilled:

$$\begin{aligned} {{\,\textrm{tr}\,}}_{A_t}\circ \mathcal {N}^{(t)}\circ \mathcal {M}^{(t)}&={{\,\textrm{tr}\,}}_{A_t}\circ \left( {{\,\textrm{tr}\,}}_K\circ \mathcal {M}_s\circ \dots \circ \mathcal {M}_{t+1}\circ {{\,\textrm{tr}\,}}_{I_t}\right) \circ \left( \,\overline{\textrm{id}}_{I_t}\circ \mathcal {M}_t\right) \\&={{\,\textrm{tr}\,}}_{A_t}\circ {{\,\textrm{tr}\,}}_K\circ \mathcal {M}_s\circ \dots \circ \mathcal {M}_t\\&={{\,\textrm{tr}\,}}_K\circ \mathcal {M}_s\circ \dots \circ \mathcal {M}_{t+1}\circ {{\,\textrm{tr}\,}}_{I_t}\\&=\mathcal {N}^{(t)}. \end{aligned}$$

Hence, Theorem 3.1 yields that there exists a CPTP map $\mathcal {D}^{(t)}:K\rightarrow K\otimes K$ such that

$$\begin{aligned} \overline{\mathcal {N}^{(t-1)}} = \mathcal {N}^{(t)}\circ \mathcal {M}^{(t)}=\left( \overline{\mathcal {M}^{(t)}}\otimes \overline{\mathcal {N}^{(t)}}\right) \otimes \mathcal {D}^{(t)}, \end{aligned}$$

where $\overline{\mathcal {M}^{(t)}}:={{\,\textrm{tr}\,}}_K\circ \mathcal {M}_t$ and $\overline{\mathcal {N}^{(t)}}:={{\,\textrm{tr}\,}}_K\circ \mathcal {M}_s\circ \dots \circ \mathcal {M}_{t+1}$. Starting from (44) and using this induction step repeatedly, we obtain

$$\begin{aligned} {{\,\textrm{tr}\,}}_K\circ \mathcal {M}_s \circ \dots \circ \mathcal {M}_1=\left( \overline{\mathcal {M}}_1\otimes \dots \otimes \overline{\mathcal {M}}_s\right) \circ \mathcal {D}, \end{aligned}$$

where $\overline{\mathcal {M}}_i={{\,\textrm{tr}\,}}_K\circ \mathcal {M}_i$ and $\mathcal {D}=\mathcal {D}^{(s-1)}\circ \dots \circ \mathcal {D}^{(1)}$ (see Fig. 6). $\square$

### Remark 4.6

In the same way as Corollary 4.1 replaces Condition (i) of Theorem 3.1 by a commutation condition, one may replace assumption (41) of Corollary 4.5 on the maps $\mathcal {M}_1, \ldots , \mathcal {M}_s$ by a commutation assumption, namely that changing the order of the maps in the concatenation $\textrm{tr}_K \circ \mathcal {M}_s \circ \cdots \circ \mathcal {M}_1$ does not have an effect. This results in a statement similar to Corollary 4.1: If the $s \ge 2$ maps on the left-hand side of (42) commute and satisfy the unitality condition, then they factorise as on the right-hand side of (42).

## Data Availability

Data sharing is not applicable to this paper as no datasets were generated or analysed during the current study.

## On the Choi-Jamiołkowski Isomorphism

Let $\Omega _{H\tilde{H}}=\smash {|\Omega \rangle \!\langle \Omega |_{H\tilde{H}}}$ be a maximally entangled state between a finite-dimensional space *H* and an isomorphic space $\tilde{H}$, which we keep fixed for the following discussion. According to Remark 2.10, we may choose an orthonormal basis $\smash {\{|i \rangle _H\}_{i \in \{1, \ldots , d\}}}$ of *H*, which then induces an orthonormal basis $\{|i \rangle _{\tilde{H}}\}_{i \in \{1, \ldots d\}}$ on $\tilde{H}$ such that

46 $$\begin{aligned} |\Omega \rangle _{H\tilde{H}}=\frac{1}{\sqrt{d}}\sum _i|i \rangle _H \otimes |i \rangle _{\tilde{H}}, \end{aligned}$$

where $$d:=\dim (H) = \dim (\tilde{H})$$.

### Definition A.1

The Choi-Jamiołkowski (C.-J.) operator of a CP map $\mathcal {M}:H\rightarrow K$ is defined as

$$\begin{aligned} \rho _{K\tilde{H}}:=\mathcal {M}(\Omega _{H\tilde{H}}). \end{aligned}$$

### Lemma A.2

Let $\rho _{K\tilde{H}}$ be the C.-J. operator of a CP map $\mathcal {M}:H\rightarrow K$. Then for all operators $W_H$ on *H*,

$$\begin{aligned} \mathcal {M}(W_H)=d^2\,\textrm{tr}_{\tilde{H}}\Big (\textrm{tr}_H\big (W_H\Omega _{H\tilde{H}}\big )\rho _{K\tilde{H}}\Big ). \end{aligned}$$

### Proof

Using Definition A.1 and (46), we can verify the claim by a direct calculation (states with the same colour yield a $\delta$-function of the corresponding labels):

$$\square $$

### Lemma A.3

A CP map $\mathcal {M}:H\rightarrow K$ is trace non-increasing if and only if its corresponding C.-J. operator $\rho _{K\tilde{H}}$ fulfils $\rho _{\tilde{H}}\le \,\overline{\textrm{id}}_{\tilde{H}}$. It is trace-preserving if and only if $\rho _{\tilde{H}}=\,\overline{\textrm{id}}_{\tilde{H}}$.

### Proof

Let

$$\begin{aligned} \mathcal {M}(W_H) = \sum _z E_z W_H E_z^* \end{aligned}$$

be the Kraus representation of $\mathcal {M}$ with Kraus operators $E_z$. We then have

$$\begin{aligned} \rho _{\tilde{H}}= \textrm{tr}_K\bigl (\mathcal {M}(\Omega _{H\tilde{H}})\bigr )&= \sum _z \textrm{tr}_K\bigl ((E_z \otimes \textrm{id}_{\tilde{H}}) \Omega _{H\tilde{H}} (E_z^* \otimes \textrm{id}_{\tilde{H}})\bigr ) \\&= \sum _z \textrm{tr}_H\bigl ( \Omega _{H\tilde{H}} (E_z^* E_z \otimes \textrm{id}_{\tilde{H}}) \bigr ) \\&= \textrm{tr}_H\bigl (\Omega _{H\tilde{H}} \sum _z E_z^* E_z \otimes \textrm{id}_{\tilde{H}} \bigr ), \end{aligned}$$

where we used the cyclicity of the trace. Because, for trace non-increasing maps, $\sum _x E_x^* E_x \le {{\,\textrm{id}\,}}_{H}$, we find

$$\begin{aligned} \rho _{\tilde{H}} \le \textrm{tr}_H\bigl (\Omega _{H\tilde{H}} ( \textrm{id}_H \otimes \textrm{id}_{\tilde{H}}) \bigr ) = \textrm{tr}_H\bigl (\Omega _{H\tilde{H}}\bigr ) = \,\overline{\textrm{id}}_{\tilde{H}}. \end{aligned}$$

The inequality becomes an equality if $\mathcal {M}$ is trace-preserving.

To show the other direction, suppose $\rho _{\tilde{H}}\le \,\overline{\textrm{id}}_{\tilde{H}}$. Then, using Lemma A.2,

47 $$\begin{aligned} \textrm{tr}_{K}\big (\mathcal {M}(W_H)\big )&=d^2\, \textrm{tr}_K\Big [\textrm{tr}_{\tilde{H}}\Big (\textrm{tr}_H(W_H\Omega _{H\tilde{H}})\rho _{K\tilde{H}}\Big )\Big ]\nonumber \\&=d^2\, \textrm{tr}_{\tilde{H}}\Big (\textrm{tr}_H(W_H\Omega _{H\tilde{H}})\underbrace{\textrm{tr}_K(\rho _{K\tilde{H}})}_{\le \,\overline{\textrm{id}}_{\tilde{H}}}\Big )\nonumber \\&\le d\, \textrm{tr}_{\tilde{H}}\big (\textrm{tr}_H(W_H\Omega _{H\tilde{H}})\big ) \nonumber \\&= d\, \textrm{tr}_H\big (W_H\underbrace{\textrm{tr}_{\tilde{H}}(\Omega _{H\tilde{H}}}_{=\,\overline{\textrm{id}}_{H}})\big )\nonumber \\&=\textrm{tr}_H(W_H). \end{aligned}$$

The inequality in (47) becomes an equality if $\rho _{\tilde{H}}=\,\overline{\textrm{id}}_{\tilde{H}}$. $\square$

### Lemma A.4

A CP map $\mathcal {M}:H_A \otimes H_B \rightarrow K_A \otimes K_B$, with $H_A \otimes H_B$ finite-dimensional, has product form $\mathcal {M}^{(A)}\otimes \mathcal {M}^{(B)}$, where $\mathcal {M}^{(A)}:H_A\rightarrow K_A$ and $\mathcal {M}^{(B)}:H_B\rightarrow K_B$ if and only if its C.-J. operator $\rho _{K_A K_B \tilde{H}_A \tilde{H}_B}$ has product form $\rho _{K_A \tilde{H}_A} \otimes \rho _{K_B \tilde{H}_B}$.

### Proof

According to Remark 2.10, the entangled state $|\Omega \rangle _{H \tilde{H}}$ used for the C.-J. isomorphism can be decomposed as

$$\begin{aligned} |\Omega \rangle _{H_AH_B\tilde{H}_A\tilde{H}_B}=|\Omega \rangle _{H_A\tilde{H}_A}\otimes |\Omega \rangle _{H_B\tilde{H}_B}, \end{aligned}$$

where $|\Omega \rangle _{H_A\tilde{H}_A}$ and $|\Omega \rangle _{H_B\tilde{H}_B}$ are maximally entangled states on the respective subsystems. Suppose now that $\mathcal {M}=\mathcal {M}^{(A)}\otimes \mathcal {M}^{(B)}$. The corresponding C.-J. operator is then given by

$$\begin{aligned} \rho _{K_AK_B\tilde{H}_A\tilde{H}_B}&=\mathcal {M}\big (\Omega _{H_AH_B\tilde{H}_A\tilde{H}_B}\big )\\&=\mathcal {M}^{(A)}\otimes \mathcal {M}^{(B)}\big (\Omega _{H_A\tilde{H}_A}\otimes \Omega _{H_B\tilde{H}_B}\big )\\&=\mathcal {M}^{(A)}\big (\Omega _{H_A\tilde{H}_A}\big )\otimes \mathcal {M}^{(B)}\big (\Omega _{H_B\tilde{H}_B}\big )\\&=:\rho _{K_A\tilde{H}_A}\otimes \rho _{K_B\tilde{H}_B}, \end{aligned}$$

hence it has product form.

For the other direction, suppose that the C.-J. operator is of the form $\rho _{K_AK_B\tilde{H}_A\tilde{H}_B}=\rho _{K_A\tilde{H}_A}\otimes \rho _{K_B\tilde{H}_B}$. The corresponding map is then given by

$$\begin{aligned}&\mathcal {M}(W_{H_AH_B})\\&\quad =d^2\,\textrm{tr}_{\tilde{H}_A\tilde{H}_B}\Big (\textrm{tr}_{H_AH_B}\big (W_{H_AH_B}\Omega _{H_AH_B\tilde{H}_A\tilde{H}_B}\big )\rho _{K_AK_B\tilde{H}_A\tilde{H}_B}\Big )\\&\quad =(\dim (H_A)\dim (H_B))^2\,\textrm{tr}_{\tilde{H}_A\tilde{H}_B}\Big (\textrm{tr}_{H_AH_B}\big (W_{H_AH_B}\Omega _{H_A\tilde{H}_A}\otimes \Omega _{H_B\tilde{H}_B}\big )\rho _{K_A\tilde{H}_A}\otimes \rho _{K_B\tilde{H}_B}\Big )\\&\quad =\Big (\dim (H_A)^2{{\,\textrm{tr}\,}}_{\tilde{H}_AH_A}\big (\Omega _{H_A\tilde{H}_A}\rho _{K_A\tilde{H}_A}\big )\otimes \dim (H_B)^2{{\,\textrm{tr}\,}}_{\tilde{H}_BH_B}\big (\Omega _{H_B\tilde{H}_B}\rho _{K_B\tilde{H}_B}\big )\Big )(W_{H_AH_B})\\&\quad =:\big (\mathcal {M}^{(A)}\otimes \mathcal {M}^{(B)}\big )(W_{H_AH_B}), \end{aligned}$$

hence it has product form. $\square$

## Necessity of the Unitality Condition

In Theorem 3.1, the unitality condition (Condition (ii); see also the slightly weaker requirement mentioned in Remark 3.3), is necessary (see Theorem 3.6). The same condition also occurs in Corollary 4.1. Its necessity can be illustrated with the following example of maps for Corollary 4.1.

Let *A*, *B*, and $H = (I, J, K)$ be classical random variables, where *I*, *J*, and *K* take values $i,j\in \{0,1\}$ and $k\in \{0,1\}^{2}\cup \{\perp \}$. Define the maps ${\mathcal {X}}:H\rightarrow A\otimes H$ and ${\mathcal {Y}}:H\rightarrow B\otimes H$ as follows.

$$\begin{aligned} {\mathcal {X}}:\hspace{10pt}&\text {if }k=\perp \text { then }a\in _R\{0,1\}; (k_1,k_2):=(a,i)\\&\text {else }a:=k_1\oplus (i\cdot k_2) \\ {\mathcal {Y}}:\hspace{10pt}&\text {if }k=\perp \text { then }b\in _R\{0,1\}; (k_1,k_2):=(b,j)\\&\text {else }b:=k_1\oplus (j\cdot k_2) \end{aligned}$$

where $a\in _R\{0,1\}$ means that *a* is chosen uniformly at random.

These maps fulfil all conditions in Corollary 4.1 except for the unitality condition: Firstly, they are CP and TP since they are defined in terms of functions of variables. Furthermore, it is clear from their definitions that $\textrm{tr}_{H} \circ {\mathcal {X}}$ and $\textrm{tr}_H \circ {\mathcal {Y}}$ are independent of *J* and *I*, respectively, i.e., Condition (iii) holds. Finally, they commute: This is obvious for the input $(i,j,k\ne \perp )$ since in this case, the maps do not change the value of *k*. For $(i,j,k=\perp )$, the output on *A* and *B* of ${\mathcal {Y}}\circ {\mathcal {X}}$ is $a=r$ and $b=r\oplus (j\cdot i)$, where *r* is a uniform random bit. Hence, *a* and *b* are both uniformly random bits with the correlation $a\oplus b=i\cdot j$. On the other hand, the output of ${\mathcal {X}}\circ {\mathcal {Y}}$ is $b=r$ and $a=r\oplus (i\cdot j)$. Again, this means that *a* and *b* are both uniform random bits with the correlation $a\oplus b=i\cdot j$. Thus, the probability distribution $\textrm{Pr}_{AB|IJK}(\cdot ,\cdot |i,j,\perp )$ is identical for ${\mathcal {Y}}\circ {\mathcal {X}}$ and ${\mathcal {X}}\circ {\mathcal {Y}}$, hence the maps satisfy Condition (i) of Corollary 4.1.

However, $\textrm{tr}_{A}\circ {\mathcal {X}}$ is not unital, i.e., uniform distributions are not mapped to uniform distributions. This can be seen from the fact that the output on *K* is never $k=\perp$, hence the probability of $k=\perp$ is zero. In particular, the probability distribution of the output *K* is never uniform. By symmetry, $\textrm{tr}_{B}\circ {\mathcal {Y}}$ is not unital, either. Hence, Condition (ii) is violated.

The question is now whether it is still possible to find a CPTP map $\mathcal {D}$ such that $\textrm{tr}_H\circ {\mathcal {Y}}\circ {\mathcal {X}}$ can be written as $(\overline{\mathcal {X}}\otimes \overline{\mathcal {Y}})\circ \mathcal {D}$ as in Corollary 4.1, even though the unitality condition is not fulfilled. This is equivalent to asking whether the input-output behaviour described above can be generated with a setup as depicted on the right-hand side of Fig. 5. Note that this setup corresponds to that of a CHSH game (i.e., a Bell test), where Alice and Bob each have local inputs *I* and *J*, respectively, and outputs *A* and *B*. Furthermore, each of them has access to one part of a bipartite quantum system $K \otimes K$, which may, for example, be prepared in a maximally entangled state. Here, Alice’s output *A* is independent of Bob’s input *J*, and Bob’s output *B* is independent of Alice’s input *I*.

Crucially, the input-output behaviour defined by $\textrm{Pr}_{AB|IJK}$ for $k=\perp$ corresponds to a PR box [8, 10, 11], whose characteristics is that it always fulfils the winning condition, $a\oplus b=i\cdot j$, of the CHSH game. However, from [13] we know that this condition can only be fulfilled with a probability $\approx 85\%$ while Corollary 4.1 would imply that it is fulfilled with certainty if it were applicable.

We conclude that the unitality condition is necessary for Corollary 4.1, i.e., it cannot be dropped without replacement. Since the corollary is an implication of Theorem 3.1, this also shows that the unitality condition is necessary for Theorem 3.1, i.e., the claim of the theorem would be wrong if Condition (ii) were dropped—a fact that also follows from our converse statement, Theorem 3.6.

## Footnotes

1 It is crucial that both agents have access to the same system *K*, which has no predefined product structure. This distinguishes the setup from standard scenarios in quantum information theory, where the relevant space typically comes with an a priori product structure (see [1] or [3] for examples).

2 Note that in this case *G* denotes the Hilbert space, *not* the space of linear operators on *G*.

3 Here, $\perp _{K'}$ denotes the CPTP map that generates the state $\perp _{K'} = |\perp \rangle \!\langle \perp |_{K'}$; see Notation 2.5.

4 The maps $\overline{\mathcal {M}}$ and $\overline{\mathcal {N}}$ are well-defined and unique; see Remark 2.8.

5 The C.-J. operators are well-defined because we will only consider maps that take a finite-dimensional input.

6 The requirement that $\mathcal {A}$ is TP does not limit the validity of this theorem as a converse statement to Theorem 3.1. Indeed, according to Remark 3.2, the map $\mathcal {M}$ that enters Theorem 3.1 is anyway TP, and hence also $\overline{\mathcal {M}}$.

7 In fact, Condition (ii) corresponds to a slightly weaker assumption, for only one of the two maps needs to satisfy the unitality requirement.

8 Tsirelson’s bound should not be confused with Tsirelson’s result [15], which we describe as Corollary 4.2.

9 Here, $\,\overline{\textrm{id}}_{I_i}$ denotes the map that creates the corresponding state, see Notation 2.5.
